# Identification of blossom-end rot loci using joint QTL-seq and linkage-based QTL mapping in tomato

**DOI:** 10.1007/s00122-021-03869-0

**Published:** 2021-06-14

**Authors:** Yasin Topcu, Manoj Sapkota, Eudald Illa-Berenguer, Savithri U. Nambeesan, Esther van der Knaap

**Affiliations:** 1 Institute of Plant Breeding, Genetics and Genomics, University of Georgia, Athens, GA 30602 USA; 2 Center for Applied Genetic Technologies Department, University of Georgia, Athens, GA 30602 USA; 3 Department of Horticulture, University of Georgia, Athens, GA 30602 USA

## Abstract

**Key message:**

**Blossom-End Rot is Quantitatively Inherited and Maps to Four Loci in Tomato**.

**Abstract:**

Blossom-end rot (BER) is a devastating physiological disorder that affects tomato and other vegetables, resulting in significant crop losses. To date, most studies on BER have focused on the environmental factors that affect calcium translocation to the fruit; however, the genetic basis of this disorder remains unknown. To investigate the genetic basis of BER, two F_2_ and F_3:4_ populations along with a BC_1_ population that segregated for BER occurrence were evaluated in the greenhouse. Using the QTL-seq approach, quantitative trait loci (QTL) associated with BER Incidence were identified at the bottom of chromosome (ch) 3 and ch11. Additionally, linkage-based QTL mapping detected another QTL, *BER3.1,* on ch3 and *BER4.1* on ch4. To fine map the QTLs identified by QTL-seq, recombinant screening was performed. *BER3.2, *the major BER QTL on ch3, was narrowed down from 5.68 to 1.58 Mbp with a 1.5-LOD support interval (SI) corresponding to 209 candidate genes. *BER3.2* colocalizes with the fruit weight gene *FW3.2*/*SlKLUH,* an ortholog of cytochrome P450* KLUH *in Arabidopsis. Further, *BER11.1,* the major BER QTL on ch11, was narrowed down from 3.99 to 1.13 Mbp with a 1.5-LOD SI interval comprising of 141 candidate genes. Taken together, our results identified and fine mapped the first loci for BER resistance in tomato that will facilitate marker-assistant breeding not only in tomato but also in many other vegetables suffering for BER.

**Supplementary Information:**

The online version contains supplementary material available at 10.1007/s00122-021-03869-0.

## Introduction

Tomato (*Solanum lycopersicum* L.) is the second most produced and consumed vegetable in the world. The demand for this vegetable has increased over the years since the produce offers wide-ranging health benefits. This growing demand has led to a steady increase in tomato production in the world, exceeding 182 million tons in 2018 (FAOSTAT 2020). Yet, this crop faces major biotic and abiotic challenges that can lead to a substantial amount of the produce being lost. Among these, physiological disorders are abiotic syndromes that affect either the whole plant or specific parts of the plant such as fruits, roots, and leaves. These disorders render the vegetable or fruit unmarketable and thus result in significant yield losses and penalized market prices (Hagassou et al. 2019; Ikeda and Kanayama 2015). As one of the most common physiological disorders in tomato, blossom-end rot (BER) alone causes serious economic losses that may reach up to 50% in this vegetable (Taylor and Locascio 2004). Just as an example, in 2018, Hickory Hill Farm in Carlton GA, USA, experienced dramatic yield losses due to BER in organically grown hybrid tomatoes that reached up to 80% (Josh Johns and Gary Shaw, personal communication, August 9, 2018). Unfortunately, the unpredictability of BER occurrence and adverse weather conditions aggravate this problem since extreme weather events are becoming increasingly more prevalent (Barickman et al. 2014; Penella and Calatayud 2018). Despite its economic importance, the underlying causes of BER are not well understood. The occurrence of BER has been primarily attributed to calcium (Ca^2+^) deficiency (Adams and Ho 1993; Raleigh and Chucka 1944). Along this vein, aberrant regulation of cellular Ca^2+^ distribution and partitioning, especially in the distal placenta, appears to be linked to BER (de Freitas et al. 2011, 2012b, 2014). Ca^2+^ plays an important role as a structural component of cell walls and membranes, and previous studies have suggested that higher concentration of Ca^2+^ in the apoplastic space (*[Ca*^*2*+^*]*_*apo*_) affects cell wall strength and stability (de Freitas et al. 2012a; Thor 2019). Just as important is the role of calcium as an intracellular secondary messenger. Therefore, Ca^2+^ concentration in the cytosol (*[Ca*^*2*+^*]*_*cyt*_) is tightly regulated as well (Clapham 2007; Clarkson et al. 1993; Kudla et al. 2010; Thor 2019). Transient, sustained, or oscillatory elevations in the *[Ca*^*2*+^*]*_*cyt*_ initiate a signaling cascade that orchestrates induction of downstream responses needed for given stimulus, such as defense and stress response gene expression, and Ca^2+^-controlled stomatal closure (Dodd et al. 2010; Ng et al. 2001). Even though a central role for Ca^2+^ in the development of BER has been postulated for many years, neither a consistent solution nor a direct link to fruit Ca^2+^ concentration has been conclusively demonstrated (Ho and White 2005). Therefore, other physiological links to the causes of BER such as reactive oxygen species (ROS) formation have recently gained prominence (Rached et al. 2018). Because of its destructive activity, excessive ROS release upon exposure of plants to stress conditions causes cell membrane lipid peroxidation, membrane leakage, and subsequently cell death, which can lead to the development of BER (Aktas et al. 2003; Rached et al. 2018; Van Breusegem and Dat 2006). Moreover, ROS production reaches a maximum, when the rate of cell expansion during fruit growth is at its maximum (Aktas et al. 2003). As a defense mechanism against ROS, plants produce antioxidants to neutralize or alleviate the negative impact of ROS. Hence, tomato varieties that feature high levels of antioxidants show resistance to BER (Rached et al. 2018). In addition to aberrant regulation of calcium and ROS, much emphasis has been placed on other physiological and genetic factors, such as accelerated fruit growth rate, phytohormones, salinity, drought, high light intensity, fruit weight and shape to explain BER (Hagassou et al. 2019; Ho and White 2005). Typically, fruit weight and elongated shape are positively correlated to BER occurrence (Ho and White 2005; Marcelis and Ho 1999). Yet not all large fruited or oval-shaped tomato varieties feature BER to the same degree. This implies that there may be genetic basis for the disorder that is hitherto unknown. Nonetheless, only a few genetic and mapping studies have been carried out for BER, despite the desire to identify resistance loci to utilize them for crop improvement (Uozumi et al. 2012). Hence, the objective of this study is to investigate the genetic basis of BER tomato. It is our expectation that these findings will ultimately provide novel knowledge about the causes of BER and to enable marker-assisted breeding not only in tomato but also in other crops that suffer from the disorder.

## Material and methods

### Plant materials and population construction

Two segregating F_2_ populations, 17S28 (*n* = 192) and 20S166 (*n* = 192), were generated by crossing BER-resistant accessions BGV007900 (*Solanum lycopersicum* var. *cerasiforme*) and BGV008224 (*S. lycopersicum* var. *lycopersicum*), respectively, with BER-susceptible accession BGV007936 (*S. lycopersicum* var. *lycopersicum*). Furthermore, a BC_1_ population (18S243, *n* = 144) was created using the susceptible accession (BGV007936) as the recurrent parent in the BGV007900 x BGV007936 F_1_. These phylogenetically closely related accessions (Razifard et al. 2020) were selected from the “Varitome” collection, and SNP data in this collection are publicly available at SGN (https://solgenomics.net/projects/varitome/). The 17S28 and 20S166 F_2_ mapping populations were, respectively, grown in Spring 2017 and Fall 2020 and included the F_1_ and parental controls. The 18S243 BC_1_ mapping population was grown in Spring 2018 without controls. Follow-up mapping populations were 19S499 (*n* = 171) and 20S74 (*n* = 192) F_3:4_ populations that were grown in Spring and Summer 2020, respectively. Only recombinant plants in the QTL interval on ch3 and ch11, respectively, were selected and grown with parental checks. All populations were grown in greenhouse where the irrigation, temperature, and supplemental light settings are Argus controlled at the University of Georgia (Athens, USA). Briefly, plants were grown in 3.79-L pots filled with a commercial potting mix (Sun Gro® Fafard® 3B Mix/Metro-Mix 830, Sun Gro Horticulture Inc, Agawam, MA) and fertilized with Nutricote controlled release fertilizer (CRF) (18N-6P-8K with 37.5 g/pot, Florikan, Sarasota, FL) and MEG-IRON V micronutrient mix (7.5 g/pot, Florikan, Sarasota, FL) following the manufacturers’ recommendations.

### BER phenotyping

**Fig. 1:**
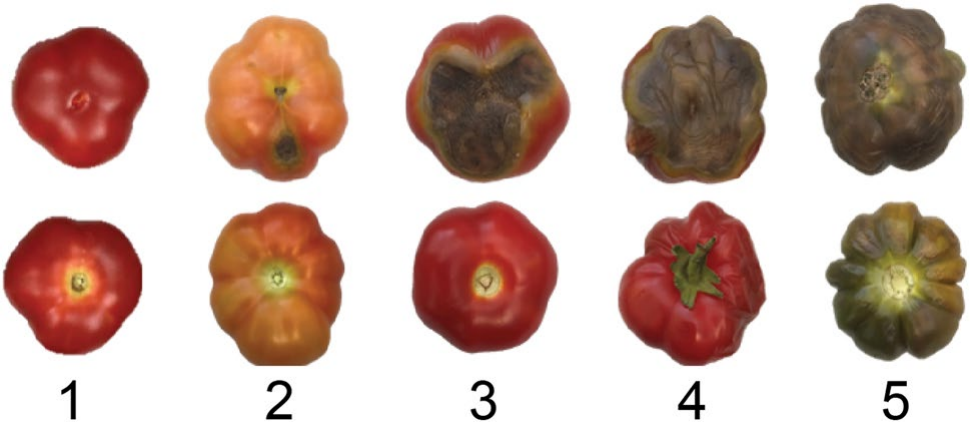
BER Visual scale from 1 to 5. As the scale number increases, the Severity of BER increases with 1 = healthy fruit with no BER symptoms and 5 = extensive BER completely affecting the fruit

To assess BER, three continuous assays and one nominal assay were developed. These assays were the following: (1) BER Severity 1, the ratio of the BER diameter to the whole fruit diameter (D_BER_/D_Fruit_); (2) BER Severity 2, the ratio of the weight of the affected part of the fruit to the total fruit weight (W_BER_/W_ALL_); (3) BER Incidence, the ratio of the number of fruit affected to the total number of fruit (AFN/TFN); and (4) BER Visual, where each fruit was scored by using a scale of 1–5 with 1 = healthy with no BER symptoms and 5 = extensive BER (Fig. 1). BER was evaluated using only the first 3–5 fruits on the first three inflorescence.

### DNA isolation and library preparation for sequencing

The DNA extraction and library preparation were performed as described before (Illa-Berenguer et al. 2015). Briefly, the genomic DNA of the plants was extracted from young true leaves using the DNeasy Plant Mini Kit (Qiagen, Valencia, CA) following the manufacturer’s recommendation. For the recombinant screening, the genomic DNA of the plants was extracted from cotyledons following the Geno/Grinder method described by Zhang et al. (2012). In the 17S28 F_2_ population, 12 plants that represented the high BER Incidence and 19 plants that represented the low BER Incidence (resistant) were selected. Similarly, 10 plants that featured high BER Severity 2 were selected for a total of three pools (Supplementary Table 1). Prior to library preparation, the genomic DNA of the plants selected for each bulk was quantified using the Qubit 2.0 Fluorimeter (Invitrogen, Carlsbad, CA, USA). For the library preparation, the NEBNext Ultra™ II DNA Library Prep Kit (New England Biolabs, USA.) and three barcoded primers from the NEBNext® Multiplex Oligos for Illumina kits (New England Biolabs, USA.) were used. Libraries were subjected to whole genome sequencing using the Illumina NextSeq 550 (300 cycles) paired-end 150-bp (PE150) flow cells at the Georgia Genomics and Bioinformatics Core at University of Georgia (Athens, GA).

### Genome sequence analysis for QTL-seq

The generated FASTQ files were merged and then assessed using the FastQC program (version 0.11.4) (Andrews 2010). Prior to further analysis, FASTQ files were filtered and trimmed using Trim Galore (version 0.4.5) for a quality value of at least 28 ( https://github.com/FelixKrueger/TrimGalore). The remaining 150-bp paired-end reads were aligned to the genomes of the inbred tomato cultivar “Heinz 1706; version SL4.0” (Sato et al. 2012) using Burrows-Wheeler Aligner (BWA) with the default parameters (Li and Durbin 2009). Average coverage for each bulk was calculated using SAM tools (Li et al. 2009). After alignment, the SAM files were converted into BAM files using SAM tools (Li et al. 2009). The BAM files were sorted and filtered using Picard software (version 2.17.4) ( https://broadinstitute.github.io/picard/). Next, the variant calling including SNP-calling was performed using Genome Analysis Toolkit (GATK) (version 3.4–0) (McKenna et al. 2010). The recommended default settings for GATK Haplotype caller were used, and SNPs with QUAL > 30 were kept (Van der Auwera et al. 2013). The final variant call format (VCF) file was converted into a tab-delimited table using the “*VariantsToTable”* function from GATK. The tab-delimited table format file was used for downstream analyses. R package “QTLseqR” was used to identify QTL (Mansfeld and Grumet 2018).

### KASP marker development and genotyping

The parental lines were genotyped for known fruit weight and shape genes (Supplementary Table 2) including *CNR (FW2.2), KLUH (FW3.2), CSR (FW11.3), OVATE (OVATE), OFP20 (SOV1), LOCULE NUMBER (LC),* and *FASCIATED* (*FAS)* (Chakrabarti et al. 2013; Ramos 2018; Rodríguez et al. 2011; Wu et al. 2018). The 17S28 F_2_ population is segregating for *FAS* and *FW3.2*, whereas the 20S166 F_2_ population was fixed for all the known fruit weight and shape genes (Table 1). Additionally, fluorescence-based KASP markers were developed using SNPs data identified from the genome sequencing data using the Primer Express® Software version 3.0.1 (Applied Biosystems, Carlsbad, CA). The Tm of two allele-specific forward primers were selected in the range of 58–61 °C (optimum: 60 °C) with minimum total GC content of 30%. Tm difference between primer pairs was set to be maximum 1 °C, and the desired product size was determined to be between 60–200 bp. Moreover, each primer had less than five repeating nucleotides in a row and was at least 25 bp in length. Next, we BLASTed each allele-specific forward primers against the SL4.0 tomato reference genome assembly in SGN (http://solgenomics.net/tools/blast/), and primers that only corresponded to the target sequence were selected. IDT oligo analyzer tool (https://www.idtdna.com/calc/analyzer) was used to test possible secondary structures, such as hairpins and primer dimers. Primer3Plus software (http://www.bioinformatics.nl/cgi-bin/primer3plus/primer3plus.cgi) was used to design the reverse primer. Finally, allele-specific primers along with common primer were tested for possible cross-dimer formation in primer pairs using multiple primer analyzer function in Thermo Scientific Web Tools (https://www.thermofisher.com/… /thermo-scientific-web-tools.html). Either FAM™ (GAAGGTGACCAAGTTCATGCT) or HEX™ (GAAGGTCGGAGTCAACGGATT) unique tail sequences were attached to the 5’ end of the allele-specific primers. The KASP markers used in this study are summarized in Supplementary Table 2. KASP genotyping was conducted in 384-well plates with a total reaction volume of 5 μL, containing 2 μL of 20–100 ng/μL genomic DNA. A total of 3 μl of the KASP PCR reaction mix was dispensed into each well using Mantis® microfluidic liquid handler (FORMULATRIX®, Bedford, MA). KASP PCR mix and PCR conditions are summarized in Supplementary Table 3. Tecan Infinite M200 Pro microplate reader (Tecan, Group Ltd., Mannersdorf, Switzerland) was used for KASP fluorescent end-point readings after the amplification. Automated genotype calling was performed using KlusterCaller software (Version 3.4.1.39, LGC Genomics LLC) using the raw data imported from Tecan microplate reader.

**Table 1:** Genotyping results of the accessions used in the study for known fruit weight and shape genes

F_2_population	Parental name	Fruit weight genes			Locule number genes		Fruit shape genes	
		*FW2.2 CNR*	*FW3.2 KLUH*	*FW11.3 CSR*	*LC WUSCHEL*	*FAS CLV3*	*OVATE OVATE*	*SOV1 OFP20*
17S28 *n* = 192	BGV007900	1	3	1	1	1	3	3
	BGV007936	1	1	1	1	3	3	3
20S166 *n* = 192	BGV008224	1	1	1	1	3	3	3
	BGV007936	1	1	1	1	3	3	3

1: derived allele; 2: resulting in large fruit or more locules, 3: Wild-type allele; resulting in small fruit or fewer locules, *n* equals the size of the population

### Linkage map construction and QTL analysis

The R/QTL (version 1.46–2, (Broman et al. 2003)) was employed to estimate genetic distances and construct genetic linkage maps. The Kosambi map function (Kosambi 1943) was used to estimate mapping distance in centimorgan (cM) by converting recombination frequencies. The logarithm of odds (LOD) scores was estimated using nonparametric interval mapping (scanone function; model = “np”) in R/QTL since the BER Incidence data does not meet the normality assumption. To declare the presence of a significant QTL, a 99% significance threshold was determined using permutation test with 1000 permutations.

### Statistical analysis

The assumption of normality was checked using the Shapiro–Wilk tests and quantile–quantile (Q-Q) plot. Significant differences were considered at *p* < *0.05*. Histograms, scatter and box plots were created in R open-source software (version 1.2.5001, (R Core Team 2019)). Pearson correlation coefficient was calculated using JMP software (version 13.2.0 (SAS Institute Inc 2017)). The broad sense heritability of each trait (H^2^) was calculated as described by Kearsey and Pooni 1996. In brief, roughly six to nine F_1_ progenies and six to nine plants from each parent were grown with the populations, and the following formula was used to estimate H^2^;

$$H^{2} = \frac{{V_{G} }}{{V_{{F_{2} }} }} = \frac{{V_{{F_{2} }} - \frac{1}{4}(V_{{P_{1} }} + V_{{P_{2} }} + 2V_{{F_{1} }} )}}{{V_{{F_{2} }} }}$$

where V_*F2*_ denotes the variation amongst F_2_ individuals, V_*P1*_ and V_*P2*_ represent the variation amongst parents and finally V_*F1*_ shows the variation amongst F_1_ plants. The phenotypic variance for F_2_ lines is due to the combination of both genetic and environmental factors. However, the phenotypic variance amongst parental lines and F_1_ progenies is due to only environmental factors.

The gene action or degree of dominance (D/A) was calculated as the ratio between dominance and additive effects. Additive effect (A) was estimated as ½ (A_1_A_1_—A_2_A_2_), where A_1_A_1_ is the mean phenotypes of homozygous BGV007900 allele and A_2_A_2_ is the mean phenotypes of homozygous BGV007936. Dominance effect (D) was estimated as A_1_A_2_ – ½ (A_1_A_1_ + A_2_A_2_). The software QTL IciMapping (Version 4.1 (Meng et al. 2015)) was used to calculate/infer D/A values. Based on the estimates of dominance effect with those of additive effect, QTL were divided into additive effect (− 0.25 ≤ d/a ≤ 0.25), incomplete or partial dominance (± 0.25 ≤ d/a ≤  ± 0.75), complete dominance (± 0.75 ≤ d/a ≤  ± 1.25), overdominance (d/a >  ± 1.25) (Tanksley 1993).

## Results

### Phenotypic evaluation of BER traits in the 17S28 F_2_ population

**Fig. 2:**
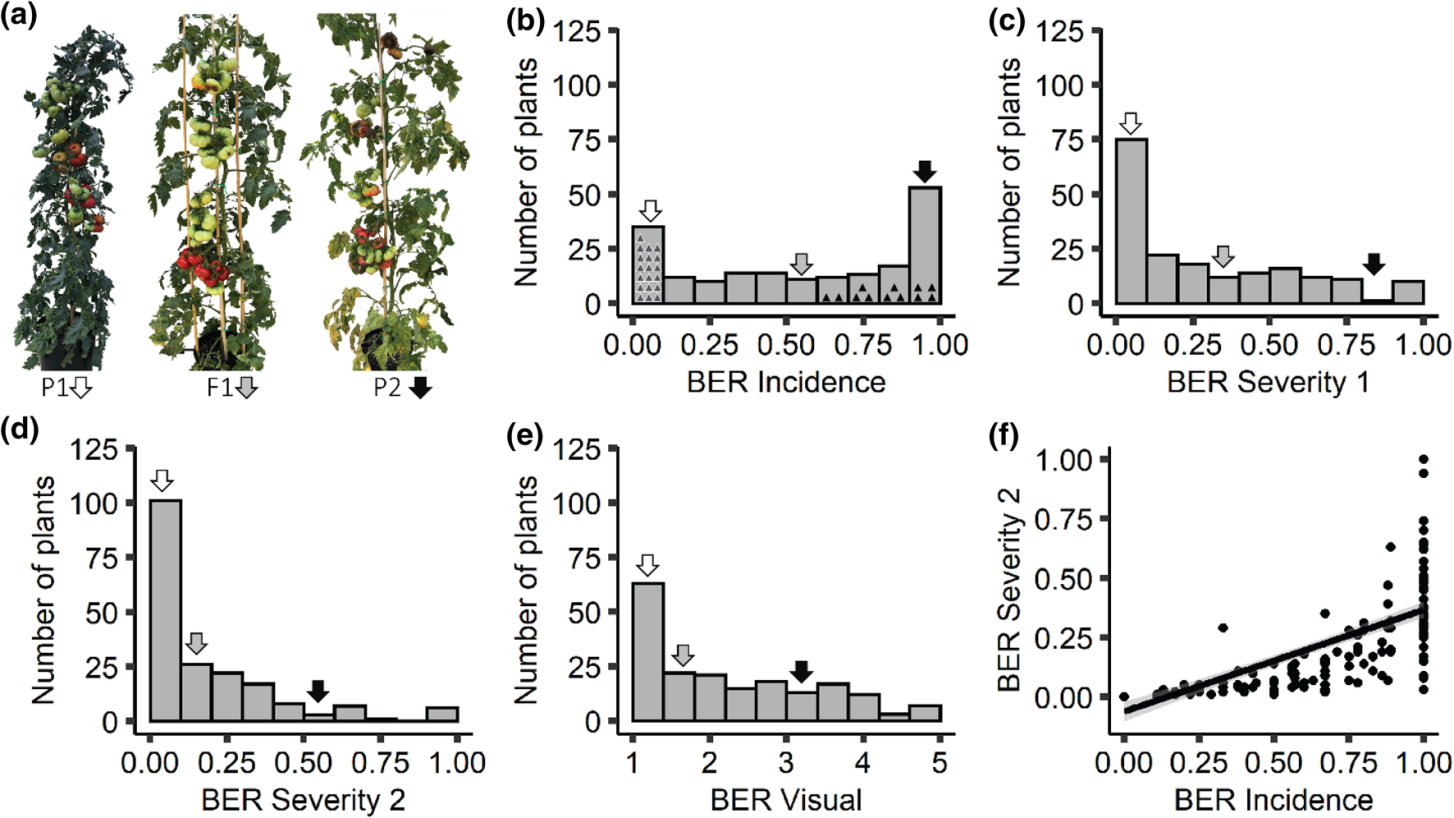
Phenotypic evaluations of BER parents and BER frequency distributions in 17S28 F_2_ population. **a** Phenotypic difference between BER-resistant parent BGV007900 (P1, with no BER Incidence), BER-susceptible parent BGV007936 (P2, with high BER Incidence), and their F_1_ generation 17S29. **b** BER Incidence frequency histogram in F_2_ progenies (17S28, *n* = 192). Gray and black triangles indicate the plants selected to generate Resistant Bulk (RB) and Susceptible Bulk (SB). White, gray, and black arrowheads on each histogram show the average of BGV007900, F_1_ and BGV007936 plants, respectively, for the trait of interest. Frequency distribution of **c** BER Severity 1, **d** BER Severity 2, and **e** BER Visual traits. **f** Correlation between BER Severity 2 and BER Incidence traits

BER was quantified using four methods: BER Severity 1 (D_BER_/D_Fruit_), BER Severity 2 (W_BER_/W_ALL_), BER Incidence (AFN/TFN), and BER Visual (a scale of 1–5). The parents of the 17S28 F_2_ population showed striking differences in terms of BER Incidence and Severity. While the resistant parent BGV007900 showed consistently low Incidence and low Severity, the susceptible parent BGV007936 displayed an opposite trend (Fig. 2a, Supplementary Fig. 1). The distribution of F_2_ plants exhibited continuous variation for each trait, indicating BER Severity 2 that the traits were quantitatively inherited (Fig. 2b-e). Yet, all distributions (BER Severity 1, BER Severity 2 and BER Visual) were skewed toward BER-resistant parent BGV007900 except for BER Incidence where the distribution was skewed toward BER-susceptible parent. The broad sense heritability for all the four BER-related traits in the 17S28 F_2_ population ranged from H^2^ = 0.48 to H^2^ = 0.80, suggesting a strong genetic basis to the BER traits (Table 2). The BER traits were also found to be significantly correlated to one another ranging from r = 0.78 to r = 0.98 (Table 3).Correlation analysis between BER Incidence and BER Severity 2 indicated that some F_2_ plants displayed high BER Incidence while showing low BER Severity 2 (Fig. 2f). This suggested that BER Incidence and BER Severity 2 may be controlled by different loci. As shown in the BER Severity 2 frequency histogram (Fig. 2d), 101 F_2_ plants were slightly affected by BER (less than 0.1 BER Severity 2). However, 6 F_2_ individuals were completely consumed by BER. A Chi-square goodness-of-fit test shows that data are consistent with a 15:1 and two segregating loci scenario (χ2_(*0.01,1*)_ = 0.075, *Prob* > *ChiSq* = *0.783* for BER Severity 2*).* With respect to BER Incidence, 48 F_2_ plants produced healthy fruits and featured less than 0.1 BER Incidence, whereas 17 plants produced fruit that were all affected by BER (Fig. 2b). The ratio of 48:17 is consistent with 3:1 and one segregating locus scenario (χ2_(*0.01,1*)_ = 0.046, *Prob* > *ChiSq* = *0.829)* for BER Incidence*.* Since we expected few loci for BER, a QTL-seq approach was used. For this purpose, genomic libraries were constructed using DNA from plants showing most extreme phenotypes from the 17S28 F_2_ population. The high BER Incidence and high BER Severity 2 plants in each pool did not overlap (Supplementary Table 1).

**Table 2:** Trait evaluations in the 17S28 F_2,_ 18S243 BC_1_ and 20S166 F_2_ populations along with parental lines

	Parent 1	F_1_	Parent 2	Mean	SD	Upper 95% mean	Lower 95% mean	Var	Min	Max	H^2^
Traits	BGV007900 * n* = 9	17S29 * n* = 9	BGV007936 * n* = 9								
*17S28 F*_*2*_* population (n* = *192)*											
BER Visual (on a 1–5 Scale)	1.00 ± 0.00b^z^	1.38 ± 0.62b	3.17 ± 0.54a	2.27	1.14	2.43	2.11	1.29	1.00	5.00	0.79
BER Incidence (AFN/TFN)	0.00 ± 0.00c	0.72 ± 0.33b	1.00 ± 0.02a	0.40	0.32	0.45	0.36	0.10	0.00	1.00	0.48
BER Severity 1 (D_BER_/D_Fruit_)	0.00 ± 0.00c	0.32 ± 0.15b	0.88 ± 0.15a	0.29	0.29	0.34	0.25	0.09	0.00	1.00	0.80
BER Severity 2 (W_BER_/W_ALL_)	0.00 ± 0.00b	0.15 ± 0.08b	0.65 ± 0.25a	0.18	0.23	0.21	0.15	0.05	0.00	1.00	0.62
*18S243 BC*_*1*_* population (n* = *144)*											
BER Visual (on a 1–5 Scale)				2.84	0.77	2.97	2.72	0.59	1.00	4.60	n.a
BER Incidence (AFN/TFN)				0.81	0.20	0.84	0.77	0.04	0.00	1.00	n.a
	BGV008224 * n* = 6	20S167 * n* = 6	BGV007936 * n* = 6								
*20S166 F*_*2*_* population (n* = *192)*											
BER Visual (on a 1–5 Scale)	1.19 ± 0.14c	3.18 ± 0.52a	4.91 ± 0.07b	3.29	0.90	3.42	3.16	0.80	1.50	5.00	0.82
BER Incidence (AFN/TFN)	0.15 ± 0.11b	0.91 ± 0.07a	1.00 ± 0.00a	0.85	0.16	0.87	0.82	0.03	0.27	1.00	0.80

*BER* blossom-end rot, *AFN* affected fruit number, *TFN* total fruit number, *D*_*BER*_ diameter of blossom-end rot scar, *D*_*Fruit*_ diameter of tomato fruit, *W*_*BER*_ weight of tissue showing blossom-end rot, *W*_*ALL*_ fruit weight of all tomato fruits evaluated. BER visual scale from 1 (with no symptoms) to 5 (severe symptoms that cover the entire fruit); Var, Variance: Min, minimum trait value; Max, maximum trait value; H^2^, broad sense heritability. Comparisons for all pairs were made using the Tukey–Kramer HSD, and levels not connected by the same letter are significantly different at α = *0.05* significance level. *n* equals the size of the population. ± Std deviation based on sample

### Identification and mapping of QTL using QTL-seq

The next-generation sequencing (NGS)-based Illumina protocol generated between 224,961,232 and 394,052,926 million 150-bp paired-end reads that after filtering were mapped to tomato reference genome (Supplementary Table 4). A total of 434,022 and 424,900 high-quality SNPs were called by comparing the BER Incidence and BER Severity 2 bulks, respectively (Supplementary Tables 5, 6). The absolute Δ(SNP index) for “SNP-index_resistant Bulk—SNP-index_ Incidence _Bulk” and “SNP-index_resistant Bulk—SNP-index_ Severity _Bulk” with a statistical confidence of *p* < *0.05* was calculated. As a result, two significant genomic positions were identified for both BER Incidence and Severity 2 traits on ch1 and ch3 (Supplementary Fig. 2,3). For BER Incidence, another genomic region was identified on ch11, whereas a different genomic position was identified for BER Severity 2 on ch8 (Supplementary Figs. 2,3). To further examine additional putative small effect QTL (the average absolute Δ(SNP-index)) was close to the 95% confidence interval), additional KASP markers were developed for ch2, ch6, ch8, and ch10 for both BER Incidence and Severity 2 traits (Supplementary Figs. 2, 3; Supplementary Table 2).

### Validation of identified QTL by SNP markers

**Fig. 3:**
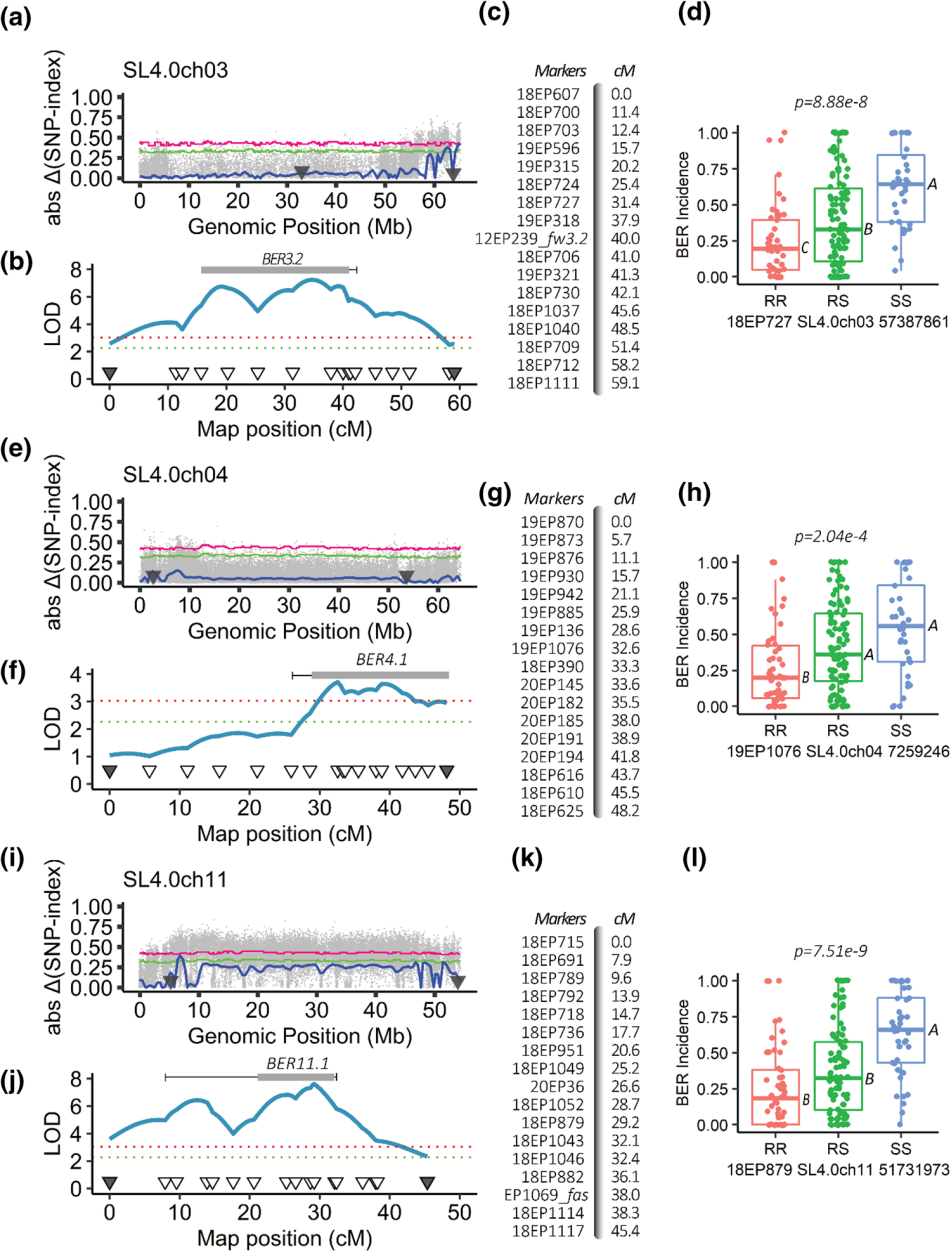
Mapping of BER Incidence in the 17S28 F_2_ population. **a**−**d** Mapping of *BER3.2*. **e**−**h** Mapping of *BER4.1*. *i*−**l** Mapping of *BER11.1*. **a, e, i** Absolute Δ(SNP-index) plots of **a** ch3, **e** ch4, and **i** ch11. X-axis shows the genomic position in Mb and Y-axis shows Absolute Δ(SNP-index). Tricube-weighted abs Δ(SNP-index) was used to facilitate visualization and interpretation of the graphs (solid blue line). *Green* and pink lines indicate the 99% and 95% confidence interval (CI), respectively, under the null hypothesis of no QTLs is present (*p* < *0.01* and *0.05*. Triangles on the X-axis show the approximate genomic positions of the first and last genotyped marker in Mb. **b, c, f, g, j, k** Linkage-based QTL mapping. Partial genetic maps and map distances of **b, c** ch3, **f, g** ch4, and **j, k** ch11. The triangles on the x-axis show the position of the genotyped markers and their corresponding genetic distances in cM, and the y-axis represents the LOD scores. A logarithm of odds (LOD) threshold of *α* = *0.01* was found at 3.05 after 1000 permutation test (red dotted line). Bars show 1.0-LOD SI, and the whiskers represent 1.5-LOD SIs for each QTL. **d, h, l** Box plots of allelic effects of the highest associated SNP markers in each QTL interval for the trait of BER Incidence. The allelic effect of **d** *BER11.1*, **h** *BER4.1,* and **l** *BER11.1.* Comparisons for all pairs were made using Tukey–Kramer HSD, and levels not connected by the same letter are significantly different at *α* = *0.05* significance level. “RR” are plants homozygous for the resistant BGV007900 allele, “RS” are plants heterozygous at this locus, and “SS” are plants homozygous for the susceptible BGV007936 allele

To validate QTL(s) identified by QTL-seq, polymorphic SNPs were converted into molecular markers to flank and encompass the identified loci. Next, marker–trait association analyses for BER Incidence and Severity 2 traits were conducted using entire 17S28 F_2_ population. Once marker–trait associations were found to be significant, additional molecular markers were developed and QTL analysis was performed. Although the QTL-seq approach found three candidate regions, only QTL at ch3 and ch11 were validated using the entire 17S28 F_2_ population (Fig. 3). KASP markers developed for BER QTL on ch1 did not show significant association with both BER Incidence and Severity 2 traits (*p* > *0.05*). Furthermore, most of the minor QTLs were not validated using marker trait association analysis except on ch4 (Fig. 3).

**Fig. 4:**
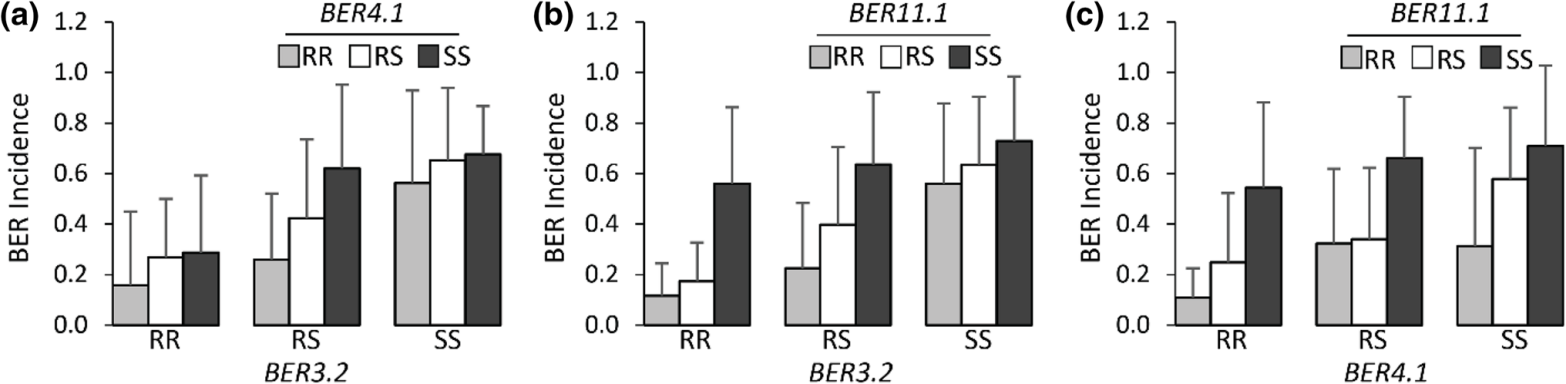
Digenic interactions of BER QTLs in the 17S28 F_2_ population. **a** *BER3.2* × *BER4.1*, **b** *BER3.2* × *BER11.1*, **c** *BER4.1* × *BER11.1.* “RR” indicates plants homozygous for the resistant BGV007900 allele, “RS” are plants heterozygous, and “SS” are plants homozygous for the susceptible BGV007936 allele

Of the two analyzed traits, BER Incidence showed the highest association with molecular markers compared to BER Severity 2. Hence, only BER Incidence was further investigated in follow-up studies. The largest effect BER QTL on ch3, *BER3.2*, accounted for 16.35% phenotypic variation and exhibited a LOD score of 7.44 (Fig. 3b). To determine the confidence intervals of the identified QTLs, *BER3.2* showed a 1.5-LOD SI extending from 15.7 to 42.1 cM (closest genetic markers 19EP596 and 18EP730, respectively), which corresponded to the physical positions of SL4.0 54,214,617 – SL4.0 59,891,210-bp (equaling 5.68 Mbp) region on the tomato reference genome of version SL4.0. Furthermore, the additive effect and D/A values for this QTL were -0.19 and 0.01, respectively. This indicated that *BER3.2* acted in an additive manner (Table 4). QTL *BER4.1* was flanked by markers 19EP885 (SL4.0ch4 5,481,420) and 18EP625 (SL4.0ch4 55,400,792) (Fig. 3f). The highest associated markers with *BER4.1* were located near the centromeric region of ch4, and 1.5-LOD SI covered nearly the entire chromosome. *BER4.1* explained a small portion of the phenotypic variance (8.57%) with a maximum LOD score of 3.74. The D/A was -0.16, suggesting that the alleles largely acted in an additive manner (Table 4). On ch11, *BER11.1* explained 17.24% of the phenotypic variation with a LOD of 8.22. *BER11.1* had a 1.5-LOD SI extending from 9.6 to 32.1 cM (closest genetic markers 18EP789 and 18EP1043, respectively), which corresponded to the physical positions of SL4.0 48,131,615—SL4.0 52,123,165 (equaling 3.99 Mbp) (Fig. 3j). The additive effect and D/A for this QTL were -0.18 and 0.38, respectively. In contrast to *BER3.2* and *BER4.1*, *BER11.1* exhibited an incomplete or partial dominance gene action for the BGV007900 allele (low BER occurrence). In addition, box plots of the highest associated SNP markers in each QTL interval are shown in Fig. 3d,h,l. Furthermore, digenic interactions of BER QTLs in the 17S28 F_2_ population were tested, but no significant epistatic or additive interactions were found among the loci (*Prob* > *F* = 0.1486 [ch3 and ch4, Fig. 4a], *Prob* > *F* = 0.5574 [ch3 and 11, Fig. 4b], *Prob* > *F* = 0.8959 [ch4 and ch11, Fig. 4c]).

**Table 3:** Pearson correlation coefficient, r, between traits in the 17S28 F_2_ population (above diagonal) and associated *p-*values (below diagonal)

r *p-value*	BER Visual (on a 1–5 Scale)	BER Incidence (AFN/TFN)	BER Severity 1 (D_BER_/D_Fruit)_	BER Severity 2 (W_BER_/W_ALL_)
BER Visual (on a 1–5 scale)	1	0.875	0.983	0.921
BER Incidence (AFN/TFN)	2.00E-61	1	0.838	0.783
BER Severity 1 (D_BER_/D_Fruit)_	2.00E-140	1.00E-51	1	0.921
BER Severity 2 (W_BER_/W_ALL_)	4.00E-79	9.00E-41	4.00E-79	1

*BER* blossom-end rot, *AFN* affected fruit number, *TFN* total fruit number, *D*_*BER*_ diameter of blossom-end rot scar, *D*_*Fruit*_ diameter of tomato fruit, *W*_*BER*_ weight of tissue showing blossom-end rot, *W*_*ALL*_ fruit weight of all tomato fruits evaluated. BER visual scale from 1 (with no symptoms) to 5 (severe symptoms that cover the entire fruit); Details regarding to BER visual score are shown in Fig. 1.

**Table 4:** Significant QTL controlling BER in the 17S28 F_2_, 18S243 BC_1_ and 20S166 F_2_ populations

Mapping population	Ch^a^	QTL^*^	Peak position (cM)	Marker interval with genetic position (cM)^b^	Physical interval (bp)^c^	LOD	PVE (%)^d^	Add^e^	D/A^f^	Prob > F (*α* = *0.05*)^g^
17S28	3	*BER3.2*	35.0	19EP596−18EP730 (15.7–42.1)	54,214,617−59,891,210	7.44	16.35	−0.19	0.01	4.704e-8
	4	*BER4.1*	32.3	19EP885−18EP625 (25.9–48.2)	5,481,420-55,400,792	3.74	8.57	−0.13	−0.16	2.101e-4
	11	*BER11.1*	29.1	18EP789-18EP1043 (9.6 - 32.1)	48,131,615–52,123,165	8.22	17.90	−0.18	0.38	8.059e-9
18S243	11	*BER11.1*	22.0	18EP951−18EP1117 (16.7–40.1)	50,569,217−54,182,901	4.62	13.75	−0.15	Na	4.773e-6
20S166*	3	*BER3.1*	6.0	20EP1015-18EP703 (0–14.1)	47,418,933–53,495,792	7.00	15.47	−0.45	0.02	1.260e-7
	4	*BER4.1*	60.0	20EP139-18EP625 (49.6–66.1)	49,843,412–55,400,792	8.85	19.12	−0.49	0.41	1.946e-9

^a^Ch Chromosome

^b^Flanking markers were defined using 1.5-LOD support interval

^c^Marker physical position based on Heinz1706 tomato reference genome (version of SL4.0)

^d^PVE Phenotypic variation explained by each QTL

^e^Add Additive effect, negative additive effect indicates that BGV007900 allele decreases BER Incidence

^f^D/A The gene action or degree of dominance of alleles

^g^the p-value of the most significant marker in the QTL interval

^*^BER3.2 (Blossom-end rot QTL on Chromosome 3 number 2)

### BER mapping in the other two populations

**Fig. 5:**
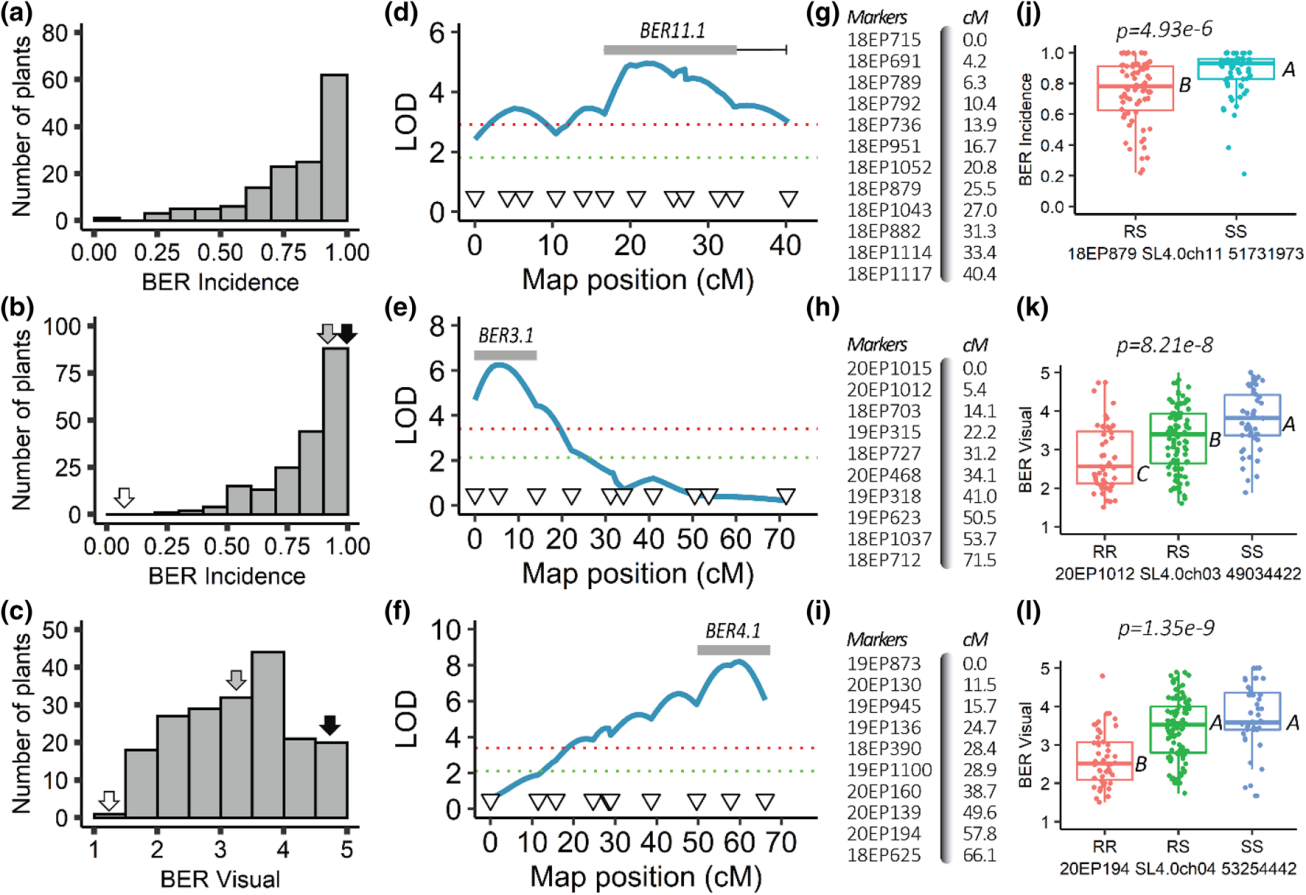
BER mapping in two additional populations. **a**−**c** Frequency distributions of BER traits. **a** BER Incidence distribution in 18S243 BC_1_, **b** BER Incidence, and **c** BER Visual distributions in the 20S166 F_2_ population. *White*, *gray*, and *black* arrowheads on each histogram show the average of BGV008224, F_1_ and BGV007936 plants, respectively, for the trait of interest. **d**−**i﻿** Linkage-based QTL mapping. Partial linkage map and map distances of **d**, **g** ch11, **e**, **h** ch3, and **f**, **i** ch4. A logarithm of odds (LOD) threshold value for *α* = *0.01* was found to be 2.98 for 18S243 BC_1_ and 3.40 for 20S166 F_2_ after 1000 permutation test. **j**−**l** Box plots of allelic effects of the highest associated SNP markers. The allelic effect of **j** *BER11.1,* **k** *BER3.1* and **l** *BER4.1.* “RR” shows plants homozygous for the resistant BGV008224 allele, “RS” plants heterozygous at this locus, and “SS” plants homozygous for the susceptible BGV007936 allele

Since most distributions in 17S28 F_2_ population were skewed toward BER-resistant parent BGV007900 and BER appeared to be additive, a backcross population 18S243 BC_1_ population was generated using the BER-susceptible BGV007936 as a recurrent parent. BER Incidence showed continuous variation in the BC_1_ population with a skewed distribution toward BER-susceptible parent BGV007936 (Fig. 5a). BER Visual also displayed continuous variation with a normal distribution (Shapiro–Wilk W test, W = 0.99, Prob < W = 0.66). Pearson correlation coefficient between BER Incidence and BER Visual was r = 0.81, indicating that both traits were highly correlated as expected. In the backcross population, *BER11.1*. has a 1.5-LOD SI extended from 16.7 to 40.4 cM (closest genetic markers 18EP951 and 18EP1117, respectively), corresponding to position SL4.0 50,569,217—SL4.0 54,182,901 bp (3.61 Mbp) (Fig. 5d,g). *BER11.1*. explained 13.75% of the phenotypic variance with a LOD score of 4.62 (Table 4). Box plots of allelic effects of the highest associated SNP marker 18EP879 are shown in Fig. 5j. In the 18S243 BC_1_ population, *BER3.2* and *BER4.1* were not segregating.

**Fig. 6:**
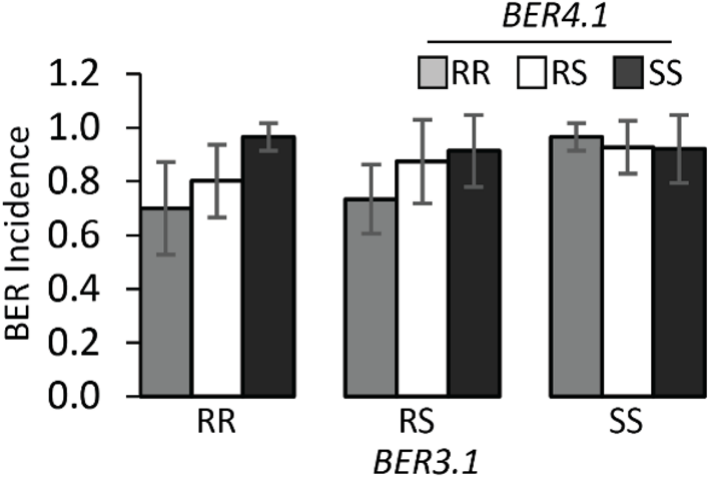
Digenic interaction of *BER3.1* × *BER4.1* in 20S166 F_2_ population. “RR” indicates plants homozygous for the resistant BGV008224 allele, “RS” are plants heterozygous, and “SS” are plants homozygous for the susceptible BGV007936 allele

Since two loci (*fw3.2* and *fas*) associated with fruit weight variation were segregating in both 17S28 F_2_ and 18S243 BC_1_ populations, a new F_2_ mapping population was developed that did not segregate for any of the known fruit weight or shape genes. The F_2_ population 20S166 was derived from a cross between BER-resistant accession BGV008224 and the same BER-susceptible accession BGV007936 (Table 1). BER Incidence distribution of F_2_ plants showed skewed distribution toward BER-susceptible parent BGV007936. A total of 187 F_2_ plants were severely affected by BER (BER Incidence ≥ 0.50) ,whereas only five plants were slightly affected by BER (BER Incidence ≤ 0.50 [Fig. 5b]). For BER Visual, F_2_ plants showed continuous variation without normal distribution (*Shapiro–Wilk W test, W* = *0.97, Prob* < *W* = *0.0011*[Fig. 5c]). Because of the extremely high BER Incidence, the BER Visual trait was used to phenotype the 20S166 F_2_ population. Using the previously identified regions linked to BER, incomplete linkage maps of ch3, ch4, and ch11 were generated, showing only two loci that segregated in the 20S166 F_2_ population (Fig. 5e,f). Interestingly, the most significant markers on ch3 did not overlap with the map position of *BER3.2* found in the other F_2_ population. Therefore, we named this locus *BER3.1* because it mapped higher on the chromosome. *BER3.1* explained 15.47% of the BER Visual variance with a LOD of 7.00 in 20S166 F_2_ population. The D/A was 0.02, suggesting that the alleles largely acted in an additive manner. The 1.5-LOD SI extended from 0 to 14.1 cM (closest genetic markers 20EP1015 and 18EP703, respectively), which corresponded to the physical interval of SL4.0ch3 47,418,933…53,495,792 bp (equaling 6.08 Mbp) (Fig. 5h). *BER4.1* explained 19.12% of the phenotypic variation with a LOD of 8.85 and thus was the most significant BER QTL in this population (Table 4). *BER4.1* had a 1.5-LOD SI extending from 49.6 to 66.1 cM (closest genetic markers 20EP139 and 18EP625, respectively), which corresponded to the physical positions of SL4.0 49,843,412—SL4.0 55,400,792 (equaling 5.56 Mbp) (Fig. 5f,i). Importantly, 1.5-LOD SI of *BER4.1* in 20S166 F_2_ population partially overlapped with the 1.5-LOD SI of *BER4.1* in 17S28 F_2_ population, suggesting that they were the same. Moreover, D/A for *BER4.1* was 0.41, indicating that *BER4.1* exhibited an incomplete or partial dominance gene action for the BGV007900 allele. Box plots of allelic effects of the highest associated SNP markers 20EP1012 and 20EP194 are shown in Fig. 5k,l. Finally, epistatic, or additive interaction between *BER3.1* and *BER4.1* was evaluated, and no significant interaction was found between the loci (*Prob* > *F* = 0.4212 [ch3 and ch4]) for the trait of BER Visual (Fig. 6).

### Fine mapping *BER3.2* and *BER11.1*

**Fig. 7:**
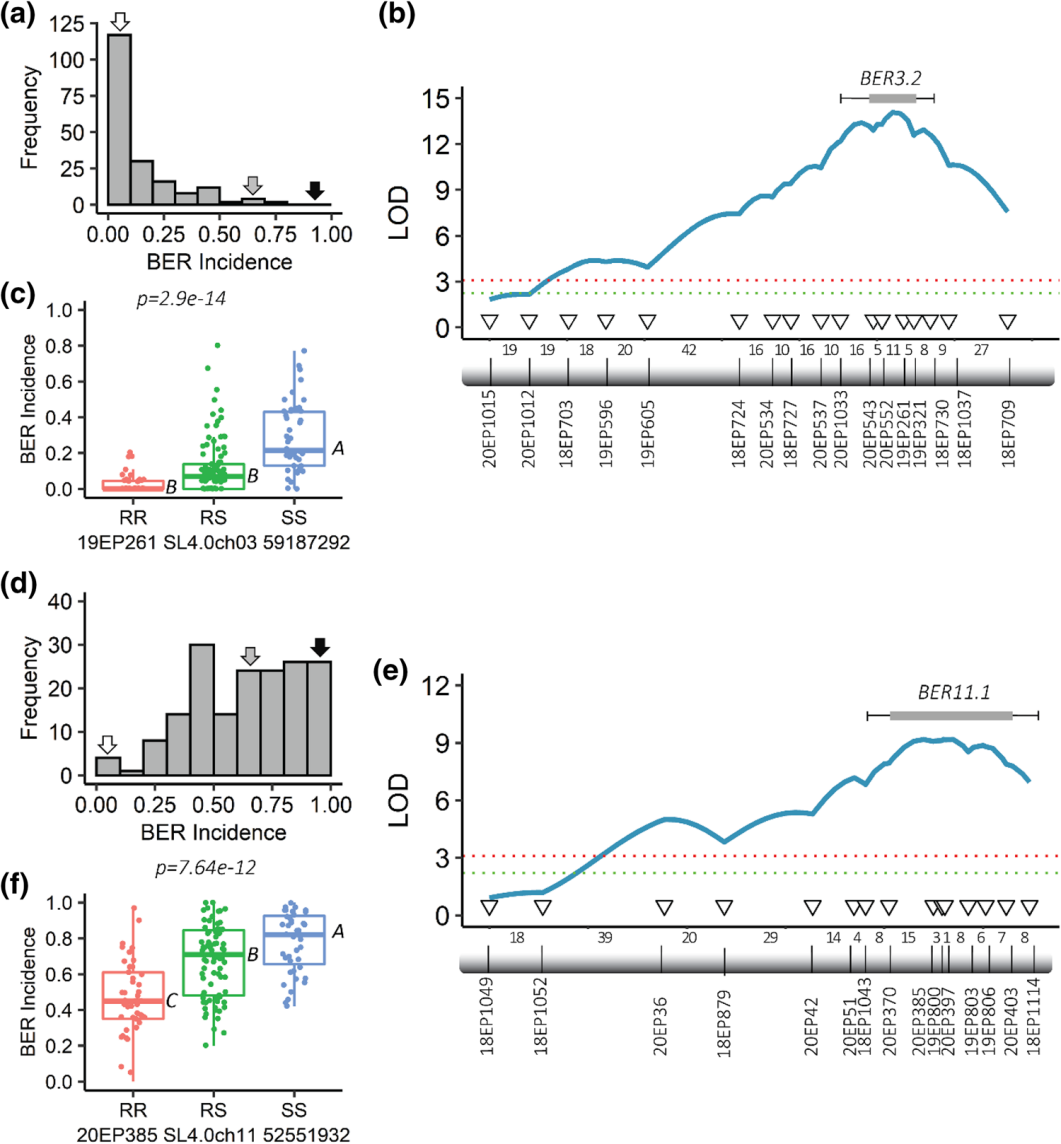
Fine mapping of the *BER3.2* and *BER11.1.* **a**−**c** Recombinant screening and fine mapping of *BER3.2* **d**−**f** Recombinant screening and fine mapping of *BER11.1* Frequency distribution of recombinant F_3:4_ plants for **a** *BER3.2* in 20S74 and **d** *BER11.1* in 19S499 F_4_ populations. *White*, *gray*, and *black arrowheads* on each histogram show the average of BGV007900, F_1_ and BGV007936 plants, respectively, for the trait of interest. **b**, **e** Linkage-based QTL mapping. **b** *BER3.2* was further delineated to approximately 1.58-Mb region flanked by 20EP1033 (SL4.0ch3 58,308,917) and 18EP730 (SL4.0ch3 59,8912,10) markers. **e** *BER11.1* was further fine mapped to 1.13-Mb region flanked by 18EP1043 (SL4.0ch11 52,123,165) and 18EP1114 (SL4.0ch11 53,258,120) markers. The numbers between genetic markers represent the number of recombinant plants. *Bars* show 1.0-LOD SI, and the whiskers represent 1.5-LOD SIs for each QTL c,f) *Box plots* of allelic effects of the most significant markers identified in the 1.5-LOD SI. The allelic effects of the most significant markers **c** 19EP2161 in *BER3.1* region and **f** 20EP385 in *BER11.1* region. “RR” indicates plants homozygous for the resistant BGV007900 allele, “RS” shows plants heterozygous at this locus, and “SS” plants homozygous for the susceptible BGV007936 allele

To further delineate the *BER3.2* and *BER11.1* intervals, recombinant screening was performed. A total of 768 F_3:4_ seedlings were genotyped with markers 18EP703 (SL4.0ch3 53,495,792) and 18EP1037 (SL4.0ch3 60,772,821) for *BER3.2* and only recombinant plants were transplanted in the greenhouse (20S74 population; *n* = 192). After the selection, the recombinant plants were genotyped with additional KASP markers. The frequency histogram of 20S74 population for the BER Incidence trait showed continuous variation with a skewed distribution toward BGV007900 resistant parent (Fig. 7a). QTL mapping showed that *BER3.2* was located between 20EP1033 (SL4.0ch3 58,308,917) and 18EP730 (SL4.0ch3 59,891,210), narrowing the locus down from 5.68 to 1.58 Mbp (Fig. 7b). This interval consists of 209 candidate genes including the fruit weight locus *FW3.2/SlKLUH*. The box plot of allelic effects of the SNP marker 19EP261 is shown in Fig. 7c.

The same approach was applied to further map *BER11.1*. A total of 1152 F_3:4_ seedlings were genotyped with markers 18EP1049 (SL4.0ch11 51,268,187) and 18EP1114 (SL4.0ch11 53,258,120), and the 19S499 population comprising of 171 recombinants were transplanted to the greenhouse. BER Incidence showed continuous variation with a skewed distribution toward BGV007936-susceptible parent (Fig. 7d). In this population, *BER11.1* mapped between 18EP1043 (SL4.0ch11 52,123,165) and 18EP1114 (SL4.0ch11 53,258,120), corresponding to a 1,134,955-bp interval (Fig. 7e). Consequently, the *BER11.1* QTL was narrowed down from 3.69 to 1.13 Mbp interval, covering 141 candidate genes. Box plot of allelic effects of the SNP marker 20EP385 is shown in Fig. 7f.

## Discussion

Despite extensive efforts to manage BER and related disorders in fruit and vegetable production, the underlying causes of this syndrome are poorly understood. While the genetic bases of physiological disorders have remained elusive, most emphasis has been placed on the physiological aspects of the syndromes. Nonetheless, these extensive efforts have delivered limited practical solutions to the problem and led to diminished production of tomato and other vegetables around the world (Taylor and Locascio 2004). Therefore, we sought to gain a better understanding about the genetic basis of BER with the expectation that these insights may lead to additional and potentially more cost-effective solutions to BER and related syndromes. In this study, we phenotyped a BER segregating population by first evaluating the methods to score the trait. Since it was expected that the disorder was quantitatively controlled and under environmental control, accurate phenotyping was deemed imperative to successfully map BER as in other complex traits (Bernardo 2020). Despite high correlations among the four traits, some F_2_ plants exhibited distinct patterns for BER Incidence and Severity 2, suggesting that these traits may be controlled by different loci. This conclusion was based on the observation that some plants carried fruits slightly affected by BER Severity 2, while exhibiting high BER Incidence values. Hence, these two traits were used to generate genomic pools for whole genome sequencing.

The QTL-seq approach has been employed to rapidly map QTL(s), and the results are often directly implemented in marker-assistant breeding (Clevenger et al. 2018; Das et al. 2015; Illa-Berenguer et al. 2015; Imerovski et al. 2019; Lu et al. 2014; Paudel et al. 2019a; Ramos et al. 2020; Wang et al. 2016). The power of QTL-seq relies on the population size, the number of SNPs between the parents and quantitative nature of the trait (Illa-Berenguer et al. 2015). In addition, the number of selected individuals in each bulk and sequencing depth also needs to be taken into consideration since these parameters significantly affect the power of QTL detection (Takagi et al. 2013). With a size of 192 plants and 778,685 SNPs that differentiate the parents, the QTL-seq approach led to the identification of *BER3.2* and *BER11.1* for the BER Incidence trait but missed the minor *BER4.1.* Therefore, traits controlled by many loci with a small effect, a higher population size (*n* ≥ 192) should be considered to capture the minor QTLs. In our study, additional markers that spanned the QTL-seq identified loci were mapped in the entire 17S28 F_2_ population, resulting in the confirmation of *BER3.1* and *BER11.1*. The other QTL on ch1 and ch8 were false positives, which is not uncommon in QTL-seq experiments (Paudel et al. 2019b). Generally for QTL-Seq, the bulk size needs to be composed of at least 15% of the total F_2_ population to detect minor QTL that explain less than 10% of the percentage of total phenotypic variation explained (Takagi et al. 2013). In this study, in an effort to include only the most extreme phenotypes, the bulks were composed of 6–10% of the total F_2_ population. This may have resulted in less power to detect the minor QTL, especially for *BER4.1* using the QTL-seq approach. *BER11.1* was validated in the BC_1_ population, whereas *BER3.2* and *BER4.1* were not. The limited size of this population may have led to an underestimation of QTL numbers in this population (Beavis 1998; Melchinger et al. 1998; Vales et al. 2005). To further confirm *BER3.2*, *BER4.1,* and *BER11.1*, we created another F_2_ population (20S166) that was not segregating for any of the known fruit weight and shape genes. In this population, we confirmed *BER4.1* and found an additional QTL on ch3 (*BER3.1*). Interestingly, *BER3.1* may have been detected in the first F_2_ population as a minor QTL close to the major QTL *BER3.2* (Fig. 3b).

We sought to refine and delineate *BER3.2* and *BER11.1*, the QTLs that were first detected and found to explain more than one third of the total phenotypic variance. *BER3.2* was narrowed down from 5.68 to 1.58 Mbp, flanked by 20EP1033 (SL4.0ch3 58,308,917) and 18EP730 (SL4.0ch3 59,891,210) markers. This region was comprised of 209 candidate genes (Supplementary Table 7) including the *FW3.2/SlKLUH* an ortholog of *KLUH* that regulates cell proliferation in developing organs in Arabidopsis (Anastasiou et al. 2007). It is known that increased fruit size and BER onset are strongly correlated despite the notion that certain large tomato varieties are resistant, whereas certain mid-sized elongated tomato varieties are susceptible to the disorder (Heuvelink and Körner 2001; Marcelis and Ho 1999). Moreover, to date, no conclusive association has been found between fruit weight genes and BER occurrence except *Cell Size Regulator* (*FW11.3*/*CSR)* that increases the fruit weight by increasing the cell size and results in high BER Incidence (Mu 2015). However, since *FW3.2/SlKLUH* was segregating in the population, we inferred that it was likely to be the gene underlying *BER3.2*. However, in addition to *FW3.2/SlKLUH*, other putative genes in this interval included *Solyc03g113920, Solyc03g113940, Solyc03g113950, Solyc03g113960, Solyc03g113970,* and *Solyc03g113980* (Supplementary Table 7) encoding calmodulin binding proteins. These proteins may be involved in Ca^2+^ signaling and ROS scavenging, which play a role in maintaining cell membrane integrity by inhibiting the cell membrane lipid peroxidation (Dhindsa et al. 1981; Van Breusegem and Dat 2006). ROS scavenging pathways are downregulated under Ca^2+^-deficient conditions, but their activities are up-regulated via Ca^2+^/calmodulin signaling (Schmitz-Eiberger et al. 2002; Yang and Poovaiah 2002; Zeng et al. 2015). In addition, other candidates included *Solyc03g114420*, a gene encoding calmodulin and *Solyc03g114110* and *Solyc03g114450*, genes related to Ca^2+^ sensing and transport (Supplementary Table 7). Hence, Ca^2+^-related genes may be considered good candidate genes for *BER3.2*. Furthermore, in the *BER3.2* interval we also found genes encoding COBRA-like proteins (*Solyc03g114880, Solyc03g114890, Solyc03g114900 and Solyc03g114910* (Supplementary Table 7) that are thought to be involved in secondary cell wall biosynthesis and fruit development. Mutations in the *COBRA-Like* genes *(COBLs)*, such as *cobl4* in Arabidopsis and *brittle stalk 2 (bk2)* in corn, affect cell wall thickness and cellulose content, especially in the sclerenchyma cells and vascular bundles (Brown et al. 2005; Ching et al. 2006). Strikingly, suppression of *SlCOBRA-like* gene in tomato leads to impaired cell wall integrity in developing fruit, coinciding with the first symptoms of BER (Cao et al. 2012). Finally, this interval included six expansion genes (*Solyc03g115270, Solyc03g115300, Solyc03g115310, Solyc03g115320, Solyc03g115340*, and *Solyc03g115345*) that may play an important role in cell wall modification and stress resistance in tomato (Lu et al. 2016; Minoia et al. 2016).

The fine mapping of *BER11.1* led to the reduction of the interval from 3.99 to 1.13 Mbp flanked by 18EP1043 (SL4.0ch11 52,123,165) and 18EP697 (SL4.0ch11 53,250,673) markers. This region was comprised of 141 candidate genes including *FASCIATED (fas),* which can affect fruit weight by controlling locule number during flower development and is due to a regulatory mutation in *SlCLV3* (Chu et al. 2019) (Supplementary Table 8). Even though *fas* segregated in the population, it is less likely a candidate for *BER11.1* since the fruit shape allele is derived from the BER-resistant parent BGV007900. We also found two pectin methylesterase genes (PMEs) (*Solyc11g070175* and *Solyc11g070187).* The role of PMEs in BER development has been studied previously, and suppression of PMEs decreases fruit susceptibility to BER (de Freitas et al. 2012a). Similar to *BER3.2*, the *BER11.1* interval contained two genes (*Solyc11g071740* and *Solyc11g071750*) encoding Ca^2+^-binding proteins. In addition to these candidates, other genes related to calcium (*Solyc11g069580*), cell division and expansion (*Solyc11g069500*, *Solyc11g069570,* and *Solyc11g069720),* and nutrient uptake (*Solyc11g069735*, *Solyc11g069750* and *Solyc11g069760*) may be plausible candidates for *BER11.1* (Supplementary Table 8)*.*

Finally, we showed that further mapping of QTLs by evaluating only the recombinant plants resulted in excellent power to detect and fine map the loci. Other methods such as progeny testing, where 10 to 20 plants derived from a single recombinant parent are evaluated, work well also but relatively few recombinants are investigated at a time. The progeny testing often results in multiple years before a locus is fine mapped to a few candidates (Chakrabarti et al. 2013; Huang and van der Knaap 2011). Therefore, space and time limitations may be met by only analyzing the recombinants near and around the locus, especially in earlier generations. Furthermore, QTL mapping can tolerate some phenotypic outliers, whereas one outlier in a progeny test can obscure the proper interpretation of the data.

To conclude, we employed the QTL-seq and linkage-based QTL-mapping approaches to genetically map four loci in three populations that were associated with BER in tomato. *BER3.2* and *BER11.1* QTL were further mapped to intervals of less than 1.6 Mb, whereas *BER3.1* and *BER4.1* await further fine mapping. The eventual cloning of the underlying genes will facilitate marker assistant breeding not only in tomato but also offer knowledge to other breeders working on vegetables and fruits that suffer from BER.

## Supplementary Information

Below is the link to the electronic supplementary material.

Supplementary file1 (DOCX 1526 kb)

Supplementary file1 (XLSX 34 kb)

Supplementary file1 (XLSX 35 kb)

Supplementary file1 (PNG 3861 kb)

Supplementary file1 (PNG 1015 kb)

Supplementary file1 (PNG 807 kb)
