# Supplementary material for: Identification of blossom-end rot loci using joint QTL-seq and linkage-based QTL mapping in tomato
Source: Theor Appl Genet. 2021 Jun 14;134(9):2931–45. doi: 10.1007/s00122-021-03869-0 (PMC8354943; doi:10.1007/s00122-021-03869-0)
Supplement: Supplementary file 1 — Supplementary file1 (DOCX 1526 kb) [file 122_2021_3869_MOESM1_ESM.docx]

**Article Title**

Identification of Blossom-End Rot Loci Using Joint QTL-Seq and Linkage-based QTL Mapping in Tomato

**Journal**

Theoretical Applied Genetics

**Authors**

Yasin Topcu^1^, Manoj Sapkota^1^, Eudald Illa-Berenguer^2^, Savithri U. Nambeesan^3^ and Esther van der Knaap^1,3*^

^1^Institute of Plant Breeding, Genetics and Genomics, University of Georgia, Athens, GA 30602, USA

^2^Center for Applied Genetic Technologies Department, University of Georgia, Athens, GA 30602, USA

^3^Department of Horticulture, University of Georgia, Athens, GA 30602, USA

*Author for correspondence ([EsthervanderKnaap@uga.edu](mailto:EsthervanderKnaap@uga.edu))

**Content: Supplementary Table 1, 3,4,5,6**

**Supplementary Table S1.** Selected F_2_ plants for susceptible and resistant bulks for QTL-seq

**Supplementary Table S3.** KASP assay mix and Thermal cycling conditions

**Supplementary Table S4.** Illumina sequencing summary for the bulks

**Supplementary Table S5.** The number of SNP between BER Resistant and BER Incidence bulks. The number of SNP polymorphism for each chromosome after filtering for reference allele frequency, minimum per sample read depth, minimum total sample read depth, maximum total read depth and minimum genotype quality.

**Supplementary Table S6.** The number of SNP polymorphism between BER Resistant and BER Severity 2 bulks. The number of SNP polymorphism for each chromosome after filtering for reference allele frequency, minimum per sample read depth, minimum total sample read depth, maximum total read depth and minimum genotype quality.

**Content: Supplementary Fig. S1,2, 3**

**Supplementary Fig. S1** Phenotypic differences between BER parents and their F_1_ generation. **a)** BER-resistant parent BGV007900 (fruits with no BER symptoms), **b)** F_1_ plant (fruits with mild and severe BER symptoms), **c)** BER-susceptible parent BGV007936 (fruits with severe BER symptoms; high BER Incidence and Severity 2).

**Supplementary Fig. S2** QTL-seq output for BER Incidence. QTL-seq applied to BER Incidence and BER Resistant bulks reveals the QTL as a peak of the average tricube smoothed abs Δ(SNP-index) value, which is showed by a solid blue line. The green and pink lines are the 99% and 95% confidence intervals under the null hypothesis of no QTLs is present (*p* < *0.01* and *0.05*).

**Supplementary Fig. 3** QTL-seq output for BER Severity 2 in tomato. QTL-seq applied to BER Severity 2 and BER Resistant bulks reveals the QTL as a peak of the average tricube smoothed abs Δ(SNP-index) value, which is showed by a solid blue line. The green and pink lines are the 99% and 95% confidence intervals under the null hypothesis of no QTLs is present (*p* < *0.01* and *0.05*).

**Supplementary Table 1.** Selected F_2_ plants for susceptible and resistant bulks for QTL-seq

| Susceptible bulk | | | | | | Resistant bulk | | |
| --- | --- | --- | --- | --- | --- | --- | --- | --- |
| **BER Incidence bulk**  **(High Incidence low Severity 2)** | | | **BER Severity 2 bulk**  **(High Incidence high Severity 2)** | | | **Resistant bulk**  **no BER** | | |
| **Plant ID** | **BER Incidence (AFN/TFN)** | **BER Severity 2 (W_BER_/W_ALL_)** | **Plant ID** | **BER Incidence (AFN/TFN)** | **BER Severity 2 (W_BER_/W_ALL_)** | **Plant ID** | **BER Incidence (AFN/TFN)** | **BER Severity 2 (W_BER_/W_ALL_)** |
| **17S28-024** | 0.74 | 0.23 | **17S28-004** | 1.00 | 0.64 | **17S28-009** | 0.00 | 0.00 |
| **17S28-039** | 0.78 | 0.19 | **17S28-015** | 0.89 | 0.63 | **17S28-011** | 0.00 | 0.00 |
| **17S28-040** | 0.87 | 0.17 | **17S28-026** | 1.00 | 1.00 | **17S28-032** | 0.00 | 0.00 |
| **17S28-042** | 0.83 | 0.30 | **17S28-106** | 0.72 | 0.70 | **17S28-035** | 0.00 | 0.00 |
| **17S28-049** | 0.95 | 0.29 | **17S28-110** | 0.88 | 0.62 | **17S28-055** | 0.00 | 0.00 |
| **17S28-051** | 0.60 | 0.07 | **17S28-115** | 1.00 | 1.00 | **17S28-068** | 0.00 | 0.00 |
| **17S28-066** | 0.63 | 0.32 | **17S28-117** | 0.50 | 0.62 | **17S28-085** | 0.00 | 0.00 |
| **17S28-095** | 0.95 | 0.26 | **17S28-127** | 1.00 | 0.65 | **17S28-121** | 0.00 | 0.00 |
| **17S28-114** | 1.00 | 0.09 | **17S28-135** | 1.00 | 0.74 | **17S28-139** | 0.00 | 0.00 |
| **17S28-120** | 0.88 | 0.29 | **17S28-186** | 1.00 | 1.00 | **17S28-142** | 0.00 | 0.00 |
| **17S28-153** | 0.75 | 0.29 |  |  |  | **17S28-149** | 0.00 | 0.00 |
| **17S28-190** | 1.00 | 0.21 |  |  |  | **17S28-151** | 0.00 | 0.00 |
|  |  |  |  |  |  | **17S28-156** | 0.00 | 0.00 |
|  |  |  |  |  |  | **17S28-161** | 0.00 | 0.00 |
|  |  |  |  |  |  | **17S28-163** | 0.00 | 0.00 |
|  |  |  |  |  |  | **17S28-172** | 0.00 | 0.00 |
|  |  |  |  |  |  | **17S28-179** | 0.00 | 0.00 |
|  |  |  |  |  |  | **17S28-180** | 0.00 | 0.00 |
|  |  |  |  |  |  | **17S28-181** | 0.00 | 0.00 |

**Supplementary Table S3.** KASP assay mix and Thermal cycling conditions

|  |  | | Volume |  | |
| --- | --- | --- | --- | --- | --- |
| KASP assay mix | Reference allele specific primer (100 µM) | | 12 µl |  | |
|  | Alternative allele specific primer (100 µM) | | 12 µl |  | |
|  | Common primer (100 µM) | | 30 µl |  | |
|  | ddH_2_O | | 46 µl |  | |
|  | **Total** | | **100 µl** |  | |
| KASP PCR reaction mix | DNA (20-160 ng/µL) | | 2 µl |  | |
|  | KASP 2x master mix | | 2.5 µl |  | |
|  | KASP assay mix | | 0.1325 µl |  | |
|  | ddH_2_O | | 0.3675 µl |  | |
|  | **Total** | | **5 µl** |  | |
| KASP PCR program | **Stages** | **Number of Cycles per stage** | **Temperature (°C)** | | **Time** |
|  | Stage 1 | 1 | 94 | | 15 min |
|  | Stage 2 | 10 | 94 | | 20 s |
|  |  |  | 65-57 | | 65 s (drop 0.8°C per cycle) |
|  | Stage 3 | 40 | 94 | | 20 s |
|  |  |  | 57 | | 60 s |
|  | Stage 4 | 1 | 10 | | ∞ |

**Supplementary Table 4** Illumina sequencing summary for the bulks

| Sample^a^ | Total Reads | Mapped reads &  % Alignment^b^ | Average  depth (X)^c^ |
| --- | --- | --- | --- |
| Resistant bulk | 224,961,232 | 214,945,730 (96.61%) | 33.94 |
| Incidence bulk | 342,460,145 | 331,808,690 (97.69%) | 52.39 |
| Severity 2 bulk | 394,052,926 | 379,942,948 (97.25%) | 59.99 |

^a^ DNA from 17S28 F_2_ individuals was sampled and bulked in each pool.

^b^ Number of reads mapped on Heinz1706 genome sequence (assembly version SL4.0). Percentage of genome covered by short reads is shown in parenthesis.

^c^ Average read depth across the genome calculated by SAM tools.

**Supplementary Table 5** The number of SNP between BER Resistant and BER Incidence bulks. The number of SNP polymorphism for each chromosome after filtering for reference allele frequency, minimum per sample read depth, minimum total sample read depth, maximum total read depth and minimum genotype quality.

| Chr | Original SNP number | Reference allele frequency ^*a^ | Minimum genotype quality ^*b^ | Minimum per sample read depth ^*c^ | Minimum total sample read depth ^*d^ | Maximum total sample read depth ^*e^ | Filtered | Remaining |
| --- | --- | --- | --- | --- | --- | --- | --- | --- |
| ch01 | 104983 | 35624 | 2139 | 71 | 82 | 13530 | 51446 | 53537 |
| ch02 | 45419 | 9953 | 642 | 112 | 90 | 1365 | 12162 | 33257 |
| ch03 | 31783 | 14378 | 664 | 51 | 53 | 1424 | 16570 | 15213 |
| ch04 | 85708 | 26519 | 741 | 104 | 90 | 1239 | 28693 | 57015 |
| ch05 | 59752 | 15341 | 498 | 55 | 70 | 1269 | 17233 | 42519 |
| ch06 | 35585 | 14732 | 468 | 83 | 60 | 2651 | 17994 | 17591 |
| ch07 | 65123 | 26593 | 527 | 85 | 54 | 1443 | 28702 | 36421 |
| ch08 | 27064 | 9337 | 546 | 245 | 132 | 1833 | 12093 | 14971 |
| ch09 | 36070 | 14421 | 485 | 51 | 55 | 2010 | 17022 | 19048 |
| ch10 | 120372 | 14096 | 1161 | 123 | 99 | 5830 | 21309 | 99063 |
| ch11 | 53858 | 19787 | 2300 | 145 | 125 | 896 | 23253 | 30605 |
| ch12 | 30390 | 12308 | 365 | 33 | 42 | 2860 | 15608 | 14782 |

^*a^ Filtering by minimum reference allele frequency: 0.2 <= REF_FRQ <= 0.8

^*b^ Filtering by minimum genotype quality GQ >= 50

^*c^ Filtering by minimum per sample read depth: DP >= 5

^*d^ Filtering by minimum total sample read depth: Total DP >= 10

^*e^ Filtering by maximum total sample read depth: Total DP <= 140

**Supplementary Table 6** The number of SNP polymorphism between BER Resistant and BER Severity 2 bulks. The number of SNP polymorphism for each chromosome after filtering for reference allele frequency, minimum per sample read depth, minimum total sample read depth, maximum total read depth and minimum genotype quality.

| Chr | Original SNP number | Reference allele frequency ^*a^ | Minimum genotype quality ^*b^ | Minimum per sample read depth ^*c^ | Minimum total sample read depth ^*d^ | Maximum total sample read depth ^*e^ | Filtered | Remaining |
| --- | --- | --- | --- | --- | --- | --- | --- | --- |
| ch01 | 105436 | 35868 | 75 | 16167 | 110 | 1967 | 54187 | 51249 |
| ch02 | 45359 | 10157 | 77 | 1540 | 108 | 645 | 12527 | 32832 |
| ch03 | 32047 | 14641 | 52 | 1715 | 50 | 871 | 17329 | 14718 |
| ch04 | 84484 | 26373 | 83 | 1372 | 114 | 825 | 28767 | 55717 |
| ch05 | 59901 | 15559 | 61 | 1561 | 87 | 505 | 17773 | 42128 |
| ch06 | 36026 | 15069 | 65 | 3417 | 75 | 722 | 19348 | 16678 |
| ch07 | 65198 | 26568 | 48 | 2014 | 88 | 479 | 29197 | 36001 |
| ch08 | 27432 | 9619 | 132 | 2022 | 216 | 707 | 12696 | 14736 |
| ch09 | 36250 | 14326 | 51 | 2628 | 49 | 522 | 17576 | 18674 |
| ch10 | 121285 | 14173 | 80 | 7632 | 135 | 2076 | 24096 | 97189 |
| ch11 | 53306 | 19855 | 100 | 1113 | 152 | 1562 | 22782 | 30524 |
| ch12 | 30770 | 12339 | 40 | 3504 | 44 | 389 | 16316 | 14454 |

^*a^ Filtering by minimum reference allele frequency: 0.2 <= REF_FRQ <= 0.8

^*b^ Filtering by minimum genotype quality GQ >= 50

^*c^ Filtering by minimum per sample read depth: DP >= 5

^*d^ Filtering by minimum total sample read depth: Total DP >= 10

^*e^ Filtering by maximum total sample read depth: Total DP <= 140


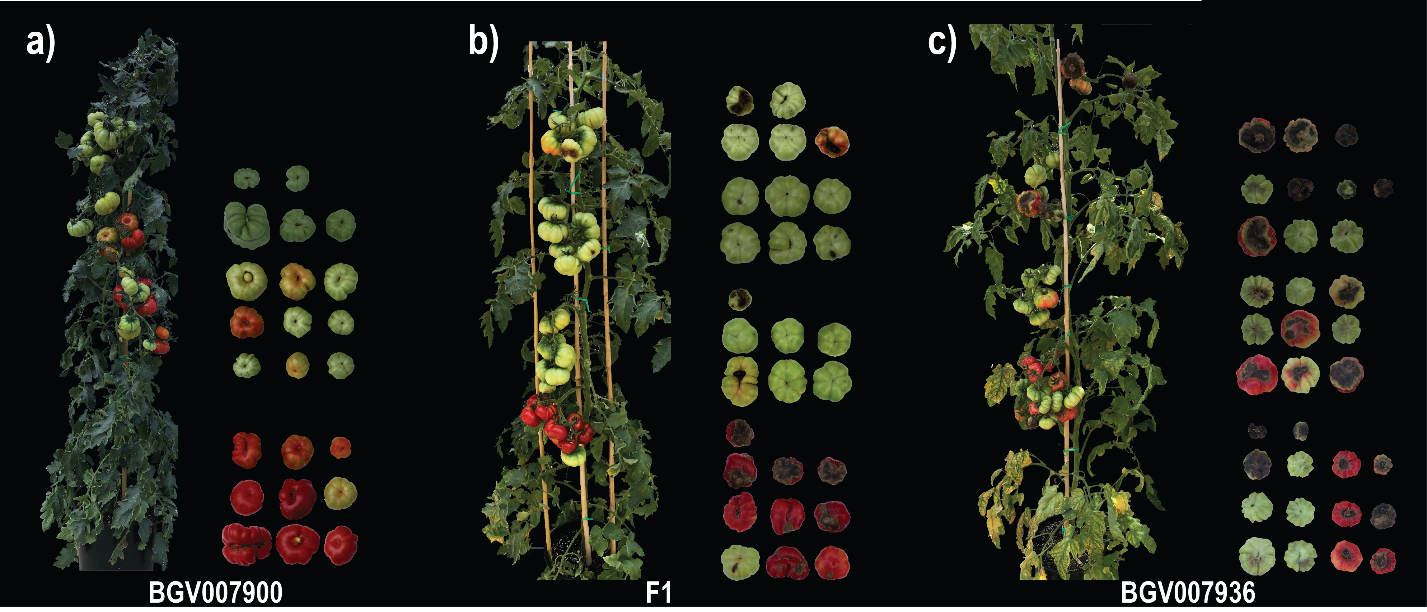


**Supplementary Fig. 1** Phenotypic differences between BER parents and their F_1_ generation. **a)** BER-resistant parent BGV007900 (fruits with no BER symptoms), **b)** F_1_ plant (fruits with mild and severe BER symptoms), **c)** BER-susceptible parent BGV007936 (fruits with severe BER symptoms; high BER Incidence and Severity 2).

**
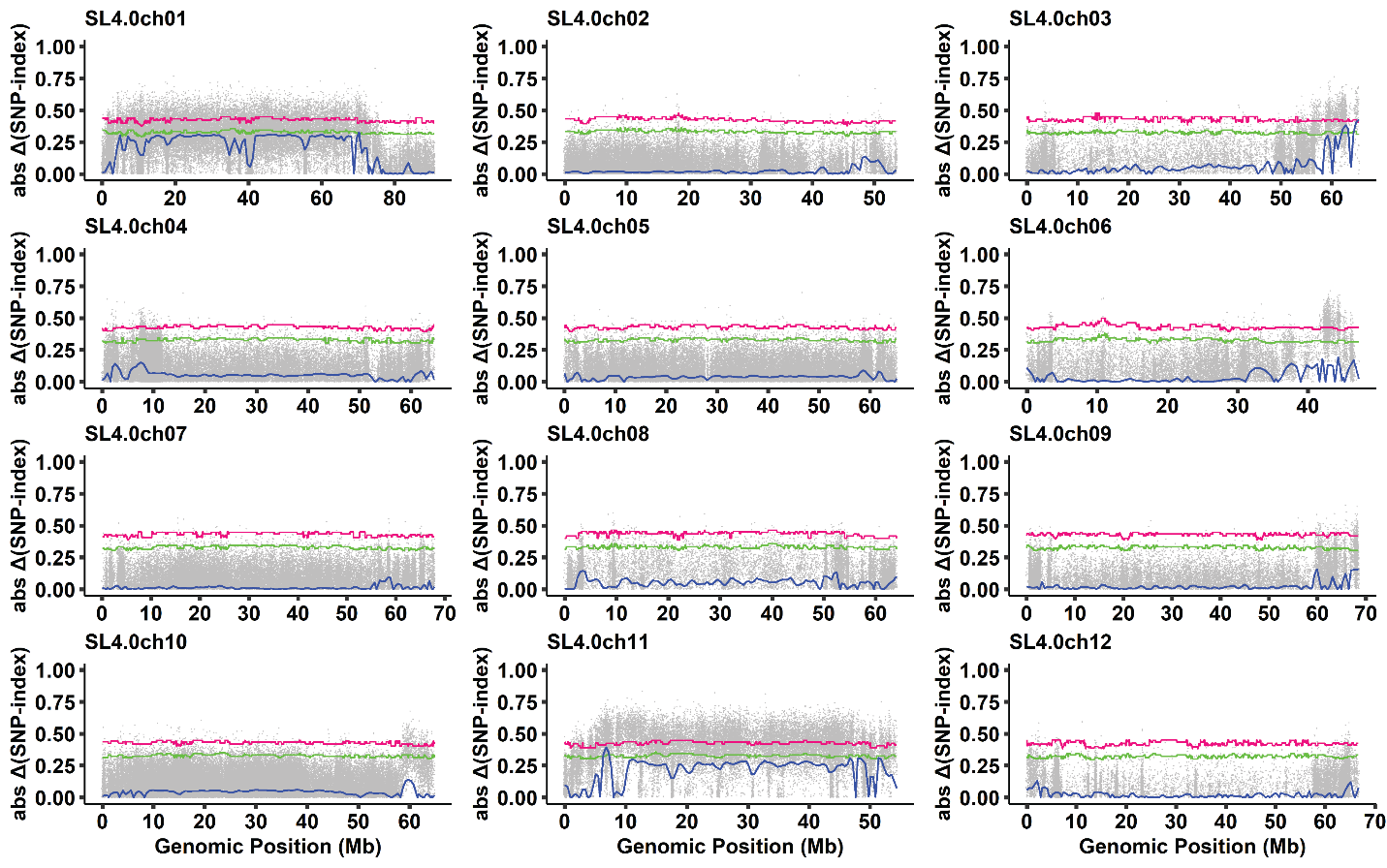
**

**Supplementary Fig. 2** QTL-seq output for BER Incidence. QTL-seq applied to BER Incidence and BER Resistant bulks reveals the QTL as a peak of the average tricube smoothed abs Δ(SNP-index) value, which is showed by a solid blue line. The green and pink lines are the 99% and 95% confidence intervals under the null hypothesis of no QTLs is present (*p* < *0.01* and *0.05*).


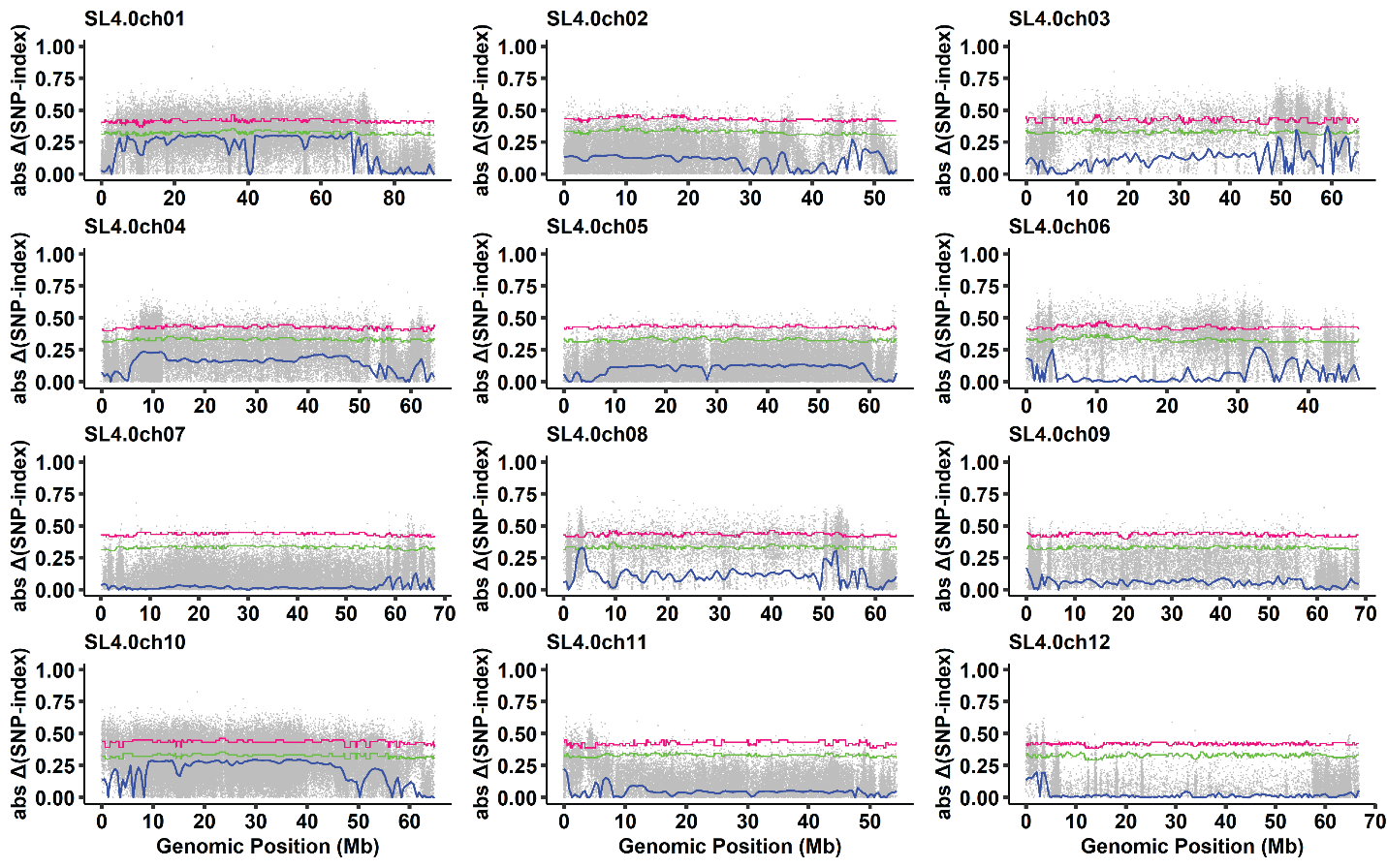


**Supplementary Fig. 3** QTL-seq output for BER Severity 2 in tomato. QTL-seq applied to BER Severity 2 and BER Resistant bulks reveals the QTL as a peak of the average tricube smoothed abs Δ(SNP-index) value, which is showed by a solid blue line. The green and pink lines are the 99% and 95% confidence intervals under the null hypothesis of no QTLs is present (*p* < *0.01* and *0.05*).
