# Supplementary figures and images for: Identification of blossom-end rot loci using joint QTL-seq and linkage-based QTL mapping in tomato
Source: Theor Appl Genet. 2021 Jun 14;134(9):2931–45. doi: 10.1007/s00122-021-03869-0 (PMC8354943; doi:10.1007/s00122-021-03869-0)

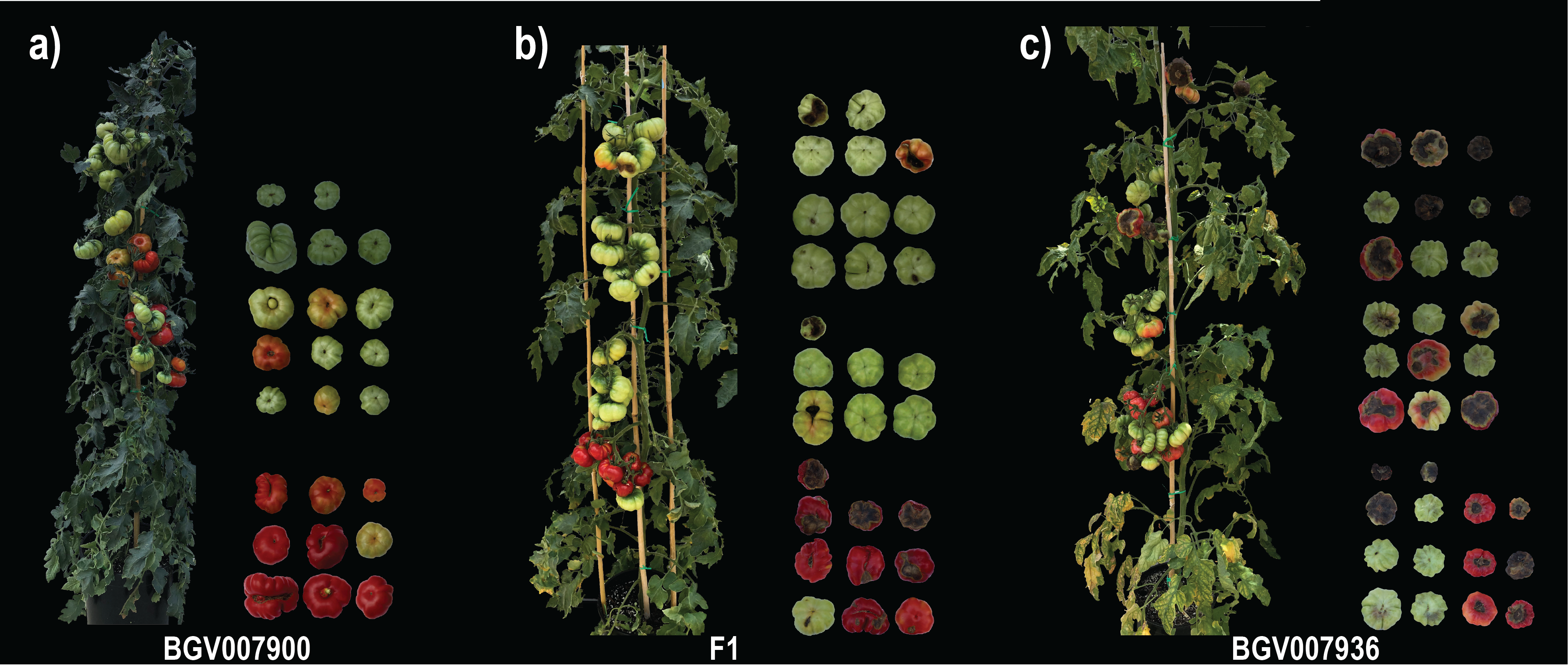

Supplement: Supplementary file 4 — Supplementary file1 (PNG 3861 kb) [file 122_2021_3869_MOESM4_ESM.png]

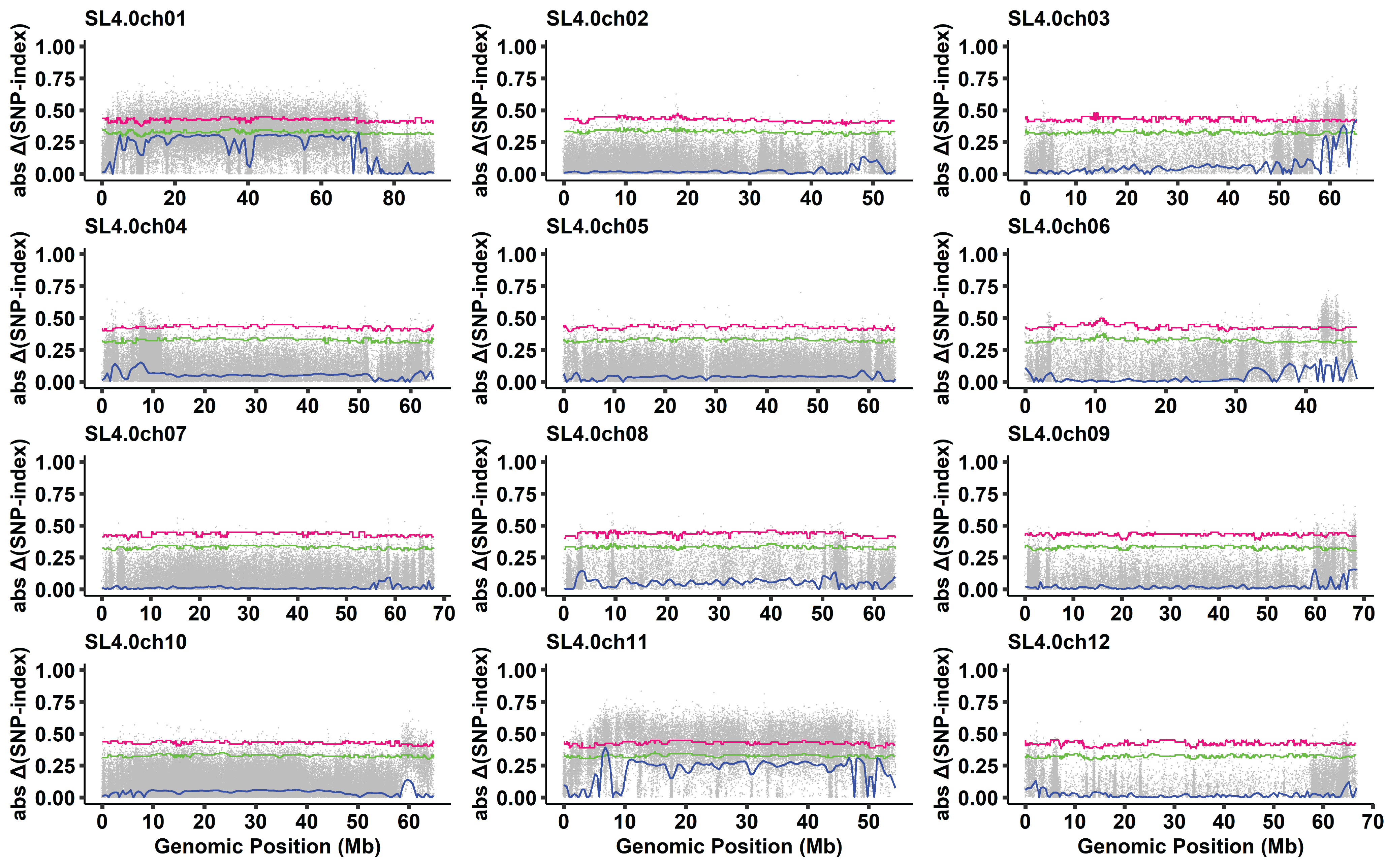

Supplement: Supplementary file 5 — Supplementary file1 (PNG 1015 kb) [file 122_2021_3869_MOESM5_ESM.png]

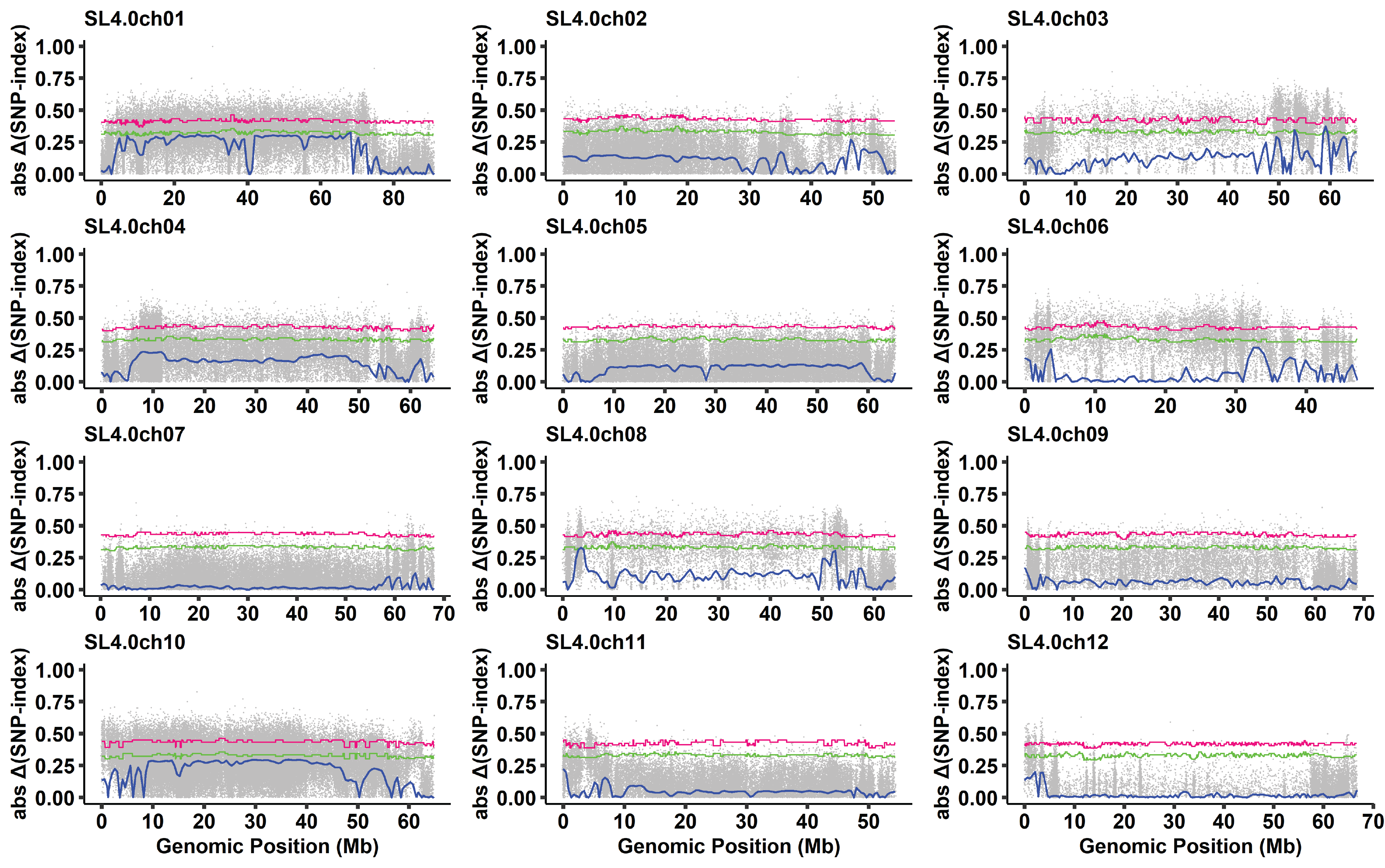

Supplement: Supplementary file 6 — Supplementary file1 (PNG 807 kb) [file 122_2021_3869_MOESM6_ESM.png]
